# Mechanosensory organ regeneration in zebrafish depends on a population of multipotent progenitor cells kept latent by Schwann cells

**DOI:** 10.1186/s12915-016-0249-2

**Published:** 2016-04-07

**Authors:** Mario Sánchez, Maria Laura Ceci, Daniela Gutiérrez, Consuelo Anguita-Salinas, Miguel L. Allende

**Affiliations:** FONDAP Center for Genome Regulation, Facultad de Ciencias, Universidad de Chile, Casilla 653, Las Palmeras 3425, Santiago, Chile

**Keywords:** Zebrafish, Regeneration, Neuromast, ErbB, Schwann cell, Lateral line

## Abstract

**Background:**

Regenerating damaged tissue is a complex process, requiring progenitor cells that must be stimulated to undergo proliferation, differentiation and, often, migratory behaviors and morphological changes. Multiple cell types, both resident within the damaged tissue and recruited to the lesion site, have been shown to participate. However, the cellular and molecular mechanisms involved in the activation of progenitor cell proliferation and differentiation after injury, and their regulation by different cells types, are not fully understood. The zebrafish lateral line is a suitable system to study regeneration because most of its components are fully restored after damage. The posterior lateral line (PLL) is a mechanosensory system that develops embryonically and is initially composed of seven to eight neuromasts distributed along the trunk and tail, connected by a continuous stripe of interneuromastic cells (INCs). The INCs remain in a quiescent state owing to the presence of underlying Schwann cells. They become activated during development to form intercalary neuromasts. However, no studies have described if INCs can participate in a regenerative event, for example, after the total loss of a neuromast.

**Results:**

We used electroablation in transgenic larvae expressing fluorescent proteins in PLL components to completely ablate single neuromasts in larvae and adult fish. This injury results in discontinuity of the INCs, Schwann cells, and the PLL nerve. In vivo imaging showed that the INCs fill the gap left after the injury and can regenerate a new neuromast in the injury zone. Further, a single INC is able to divide and form all cell types in a regenerated neuromast and, during this process, it transiently expresses the *sox2* gene, a neural progenitor cell marker. We demonstrate a critical role for Schwann cells as negative regulators of INC proliferation and neuromast regeneration, and that this inhibitory property is completely dependent on active ErbB signaling.

**Conclusions:**

The potential to regenerate a neuromast after damage requires that progenitor cells (INCs) be temporarily released from an inhibitory signal produced by nearby Schwann cells. This simple yet highly effective two-component niche offers the animal robust mechanisms for organ growth and regeneration, which can be sustained throughout life.

**Electronic supplementary material:**

The online version of this article (doi:10.1186/s12915-016-0249-2) contains supplementary material, which is available to authorized users.

## Background

The capacity to regenerate organs or tissues after injury is an intrinsic property of all organisms and varies between different species. Whereas in fish this capability is preserved from larval stages to adulthood [1–3], in other vertebrates, such as the frog, it is present only in larval stages [4, 5] or is almost completely lost, as in mammals. After injury, different organisms can thus respond either through regeneration (functional and structural restoration of the organ or tissue) or repair (replacement of the structure by another that allows survival of the organism) [6]. This balance depends on the cellular composition of the affected tissue, the physiological context, and the age of the individual [7]. It has been shown that the ability of progenitor cells to be activated and to differentiate at the injury site tilts the balance towards regeneration [7, 8]. However, the cellular and molecular mechanisms that govern the maintenance and differentiation of progenitor populations and how regeneration is triggered and controlled at the injury site are poorly understood. Recently, the lateral line of the zebrafish has emerged as a powerful model for studying the interactions between cells extrinsic or intrinsic to the organ during development and regeneration [9–13].

The lateral line is a mechanosensory system located on the surface of the fish. It is able to detect and localize water movements around the body surface and is implicated in several behaviors, such as navigation, schooling rheotaxis, and predator avoidance [14, 15]. This system is anatomically divided into an anterior lateral line, located on the head, and the posterior lateral line (PLL), distributed along the trunk and tail of the fish. The vast majority of the knowledge about this sensory system comes from the study of its posterior component. The PLL of zebrafish arises from a migratory primordium (PrimI) that, during late embryonic stages, deposits seven or eight sensory organs called neuromasts linked by a single cell-wide line of interneuromastic cells (INCs) [16]. Primary neuromasts are named by their position, from L1 (the most anterior one) to L8. Each neuromast is composed of a central core of hair cells surrounded by mantle cells, supporting cells, and progenitor cells, and is innervated by the peripheral projections of afferent neurons located in the PLL ganglion [17–20]. Together, these peripheral projections form the PLL nerve (PLLn).

The number of neuromasts increases during larval development from the initial eight, arranged in a single anteroposterior line, to over 60, distributed in four lines [21]. The increase in neuromast number is made possible by three mechanisms [21–23]. The first mechanism involves the migration of a secondary primordium (PrimII), which travels from the head to the anus depositing additional neuromasts (called secondary neuromasts) adjacent to the primary ones [21, 23, 24]. The second process is the formation of intercalary neuromasts. The intercalary neuromasts arise from primary INCs that are displaced ventrally from the horizontal myoseptum—where they were originally deposited—by the deposition of secondary neuromasts. The displacement separates the INCs from the underlying Schwann cells (SCs), stimulating their proliferation and differentiation into new neuromasts [16, 21, 24–26]. The first intercalary neuromast appears between L1 and L2, and they progressively fill all of the intersomitic borders between L1 and L2 that were left vacant by the secondary neuromasts [21]. The third and final process of PLL development involves the formation of dorsoventral columns or “stitches” of neuromasts that arise from a previously established neuromast by a process akin to budding [21, 22, 27].

It has been shown that SCs associated with the PLLn have a key role during the entire developmental process of lateral line formation [16, 25, 26]. These glial cells have the capacity to inhibit the proliferation of INCs at short range and thus prevent early formation or ectopic development of intercalary neuromasts [16, 25, 26]. Preventing SC development in the lateral line either genetically (*Sox10* or *ErbB* signaling mutants) or physically (ablation of the lateral line nerve) produces an early activation of the INCs and therefore precocious intercalary neuromast formation [16, 25, 26, 28, 29]. However, the signaling pathway involved in this process is still largely unknown [25].

Over the last decade, the PLL has become an extensively used model for regeneration and tissue homeostasis studies [9–13]. Several groups have shown that exposure of zebrafish larvae to micromolar concentrations of heavy metals like mercury [30] and copper [31–33] or to neomycin [10] kill lateral line hair cells, and that these cells reappear robustly 24 to 36 hours post injury (hpi) [13]. Not all types of damage are followed by the same outcome, however. Moderate chemical or physical injury to the fish is followed by a rapid loss of only the hair cells, without eliminating other neuromast cells, and is followed by rapid regeneration of the hair cells [5, 6]. In contrast, when zebrafish larvae are exposed to high concentrations of copper (≥100 μM), the neuromasts are entirely destroyed and no regeneration occurs [31, 33]. This result and others have revealed the presence of progenitor cells in neuromasts that can provide an inexhaustible supply of new hair cells [34]. Adult zebrafish show the same robust regeneration of hair cells as larvae after similar treatment. There is additional evidence supporting the existence of a multipotent progenitor that can give rise not only to hair cells, but to all of the cell types of a neuromast. For instance, if the adult tail fin is cut, the remaining lateral line cells are able to proliferate and invade the regenerated tail, forming new neuromasts [9]. These observations, however, leave open the question regarding the cellular mechanisms involved in the restoration of an entire neuromast after the removal of all cells and how coordination of cellular behaviors favors a regenerative response.

Here, we address this question by using electroablation [35] to eliminate all of the cells of a single neuromast and follow the behavior of remaining lateral line cells. By combining genetic labeling with cell lineage experiments, we show that INCs are dormant multipotent progenitor cells distinct from precursor cells that reside within the neuromasts. After neuromast damage, the INCs located adjacent to the injury site have the ability to migrate into the gap, proliferate, and differentiate in order to form a new and complete sensory organ. We also show that the regenerated organs are chimeric structures derived from at least two interneuromastic progenitor cells. Importantly, we find that regeneration in this context is highly dependent on an inhibitory factor produced by SCs, most likely the same factor that acts during development to limit the production of sensory organs to specific locations along the body.

## Results

### Single neuromast electroablation locally ablates all components of the PLL system

Our aim was to understand the cellular mechanisms involved in the activation and differentiation of sensory organ precursor cells after complete neuromast ablation. To this end, we have taken advantage of a simple methodology recently developed in our laboratory, electroablation [35]. This technique allowed us to induce a localized tissue injury by applying an electrical pulse directly to the neuromasts, which are superficially located, to completely remove them.

We decided to ablate the third neuromast (L3) of the PLL in larvae 3 days post fertilization (dpf) because, at this stage, the primary PLL is completely laid down and innervated [18]. Also, the region where L3 is located is easily recognizable and no intercalary neuromasts appear normally in its vicinity at the stages examined [21], avoiding potential misinterpretation of the results or interference by normal developmental processes in our observations. Furthermore, the secondary primordium (PrimII) does not travel as far caudally as L3 and does not generate secondary neuromasts near it.

**Fig. 1:**
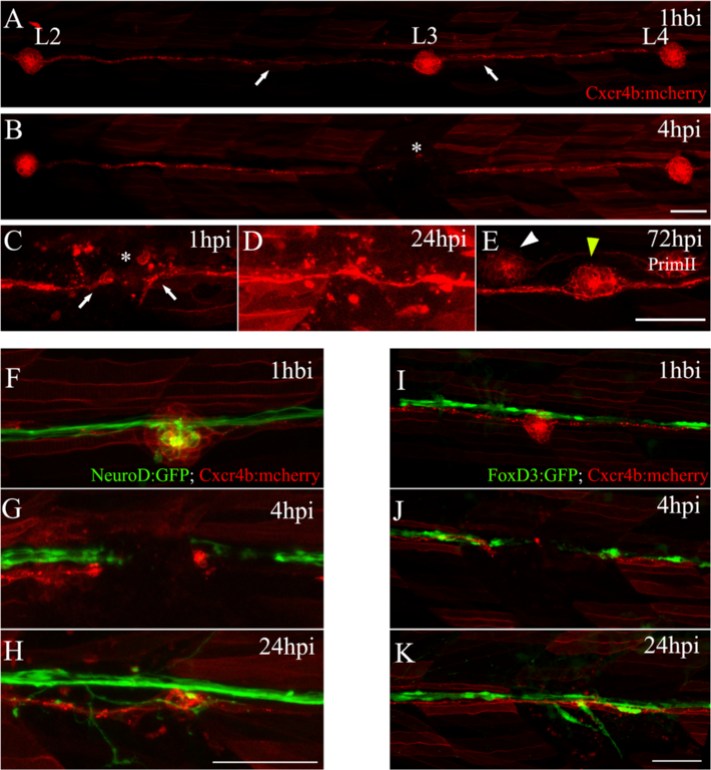
Electroablation as a method for localized tissue injury in the posterior lateral line (PLL) of zebrafish larvae. **a** Trunk of a *tg*(*cxcr4b:mCherry*) larva showing *red-labeled* PLL cells, including the second, third, and fourth neuromasts of the PLL (*L2*, *L3*, and *L4*) connected by interneuromastic cells (INCs, *white arrows*). The image was captured 1 hour before injury (*hbi*). **b** The trunk of the same larva 4 hours post injury (*hpi*). The *asterisk* shows the damaged zone, where the L3 neuromast was located. **c**–**e** Higher magnifications of the injured area showing the process of neuromast regeneration. **c** At 1 hpi, we observed the gap generated between INCs (*white arrows*) flanking the injury zone (asterisk). **d** At 24 hpi, INCs located on both sides of the gap reconnected. **e** At 72 hpi, the L3 neuromast had regenerated (*yellow arrowhead*). At this stage, the secondary primordium (*PrimII*) deposited a secondary neuromast (*white arrowhead*). **f**–**h** Double transgenic *tg*(*neurod:GFP; cxcr4b:mCherry*) larvae, where the afferent lateral line neurons are labeled in *green* and neuromasts are labeled in *red*. **f** This image shows the L3 region 1 hbi. **g** Electroablation of L3 interrupts the lateral line nerve. **h** The nerve regenerates after 24 hpi. **i**–**k** Double transgenic *tg*(*foxd3:GFP; cxcr4b:mCherry*) larvae, showing the Schwann cells labeled in *green* (associated with the nerve) and neuromasts and INCs labeled in *red*. As occurs with the INCs, Schwann cells reconnected after 24 hpi (**k**). Scale bar: 50 μm

In order to ablate the L3 neuromast, we applied two 8 μA pulses for 2 s each directly over the neuromast in *tg*(*cxcr4b:mCherry*) transgenic larvae; in this line, PLL neuromasts and INCs express membrane-tagged red fluorescent protein (RFP) [36]. As shown in Fig. 1a, intact trunk neuromasts (L2, L3, and L4 are shown) have a rosette-like structure and are interconnected by INCs (Fig. 1a, arrows). Figure 1b shows the trunk of the same larva 4 hpi. As was previously reported [35], our electroablation protocol creates a gap of 55.8 ± 26.3 μm between remaining INCs at the position where L3 was located, with no remaining primordium-derived cells in the gap (Fig. 1b, asterisk; 1c). In every experiment, we confirmed that this was the case before proceeding. Adjacent neuromasts L2 and L4, as well as most of the surrounding INCs, remained intact. After 24 hpi, surviving lateral line cells migrated and converged midway to seal the gap (Fig. 1d). After 72 hpi, a new L3 regenerated at the same position as the original L3 in 58.2 ± 4.4 % of cases (Fig. 1e, yellow arrowhead; Additional file 1). Thus, electroablation can be used to inflict localized—yet complete—damage to a single neuromast and to study its regeneration.

To further characterize the impact of electroablation on the underlying lateral line components, such as the PLLn, we repeated the same experiment in double transgenic *tg*(*neurod:GFP; cxcr4b:mCherry*) larvae that express cytoplasmic green fluorescent protein (GFP) in the axons of the lateral line afferent neurons and mCherry in all cells derived from PrimI (Fig. 1f). Ablation of the L3 neuromast produced a complete interruption of the PLLn (Fig. 1g). Between 3 and 9 hpi, the distal nerve degenerated (Additional file 2, white arrowheads) as previously described [37, 38]. As a consequence of distal nerve degeneration, caudal neuromasts (L4–L8) were temporarily deprived of afferent innervations, whereas the neuromasts located rostral to the damaged site (L1, L2) remained innervated. After 9 hpi, the axons began to regenerate (Additional file 2). As a consequence of the sprouting behavior of the regenerating nerve, a few afferent axons defasciculated and their growth became arrested at the gap left by electroablation (Fig. 1h; Additional file 2, yellow arrowhead). Most axons, however, grew along the myoseptum (Additional file 2C, F, yellow arrow), reaching the tip of the tail and reinnervating neuromasts caudal to L3 (Additional file 2C, F, white arrow). Nerve regeneration ended after 30 hpi, and INCs had sealed the breach created by ablation of the L3 neuromast.

At 3 dpf, the PLLn is lined by SCs that will form the myelin sheath [29]. We presumed that the loss of the neuromast and nerve caused by electroablation would be accompanied by a local loss of SCs in the damaged zone. Previous work by ourselves and others has shown that SCs undergo significant changes (dedifferentiation and/or cell death) when the nerve they are interacting with disappears [38–44]. We examined the fate of SCs in our conditions by using double transgenic *tg*(*foxd3:GFP; cxcr4b:mCherry*) larvae that express GFP in the SCs and RFP in the lateral line components (Fig. 1i). As we observed with the PLLn, SCs located under the L3 neuromast were completely eliminated, generating a temporary gap that was filled after 24 hpi (Fig. 1i–k). Thus, glial cells and INCs sealed the injury zone at approximately the same time. However, the differentiation process of SCs was reversed after temporary denervation, as was previously reported in several animal models including zebrafish [39, 40, 45, 46]. Whereas control 3 dpf zebrafish larvae started to express myelin basic protein (MBP), which is a differentiation marker of myelinating cells [44] (Additional file 3A, arrowheads), in injured larvae we observed a fragmentation and then loss of MBP expression caudal to L3 (compare Additional file 3C, D with I–J). By 24 hpi, there was a complete absence of the MBP marker from the injury point to the caudal fin (Additional file 3B; also compare Additional file 3E, F with K, L). The loss of MBP expression, but not of the Foxd3:GFP signal, after 24 hpi suggests changes in the differentiation state of SCs rather than death of these cells. After 48 hpi, we observed a partial recovery of MBP expression in the caudal zone, as was previously reported [38], suggesting that the myelination process had restarted after nerve regeneration.

Thus, we have shown that the continuity of the PLLn, SCs, and INCs along the PLL is interrupted by electroablation at the position of neuromast L3. Further, we show that all three components (neuromast/INCs, axons, and SCs) reestablish continuity of the PLL after a few days, indicating a coordinated regeneration process. We then sought to examine the behavior of these cells and determine how they contribute to the regeneration of L3 after its ablation.

### Identification of the cells that contribute to a regenerated neuromast

In both axolotl and zebrafish, remaining neuromasts contribute to the regeneration of the lateral line after tail fin amputation [9, 47]. To evaluate if this mechanism could account for neuromast regeneration in our model, we looked for changes in the number of cells in neighboring neuromasts after damage to L3. To that end, we counted the hair cells in the L2 and L4 neuromasts by in vivo observation of *tg*(*brn3c:GAP43-GFP*) L3 electroablated larvae 2, 24, 48, and 72 hpi. We also fixed ablated and control larvae at several time points after injury (6, 24, 48, and 72 hpi) and carried out immunohistochemistry to identify and quantify additional cell types in the L2 and L4 neuromasts (Additional file 4). Total cell numbers were quantified by TO-PRO-3 (nuclear staining) while ET20:GFP transgenic larvae in combination with Sox2 immunolabeling were used to recognize mantle cells (GFP^+^Sox2^+^) and the progenitor cell population (GFP^−^Sox2^+^) [33, 48]. We could not detect any significant differences in the cell composition of the L2 and L4 neuromasts when compared to the control and injured animals. We therefore concluded that neighboring neuromasts were not affected by L3 damage and that progenitor cells residing in neighboring neuromasts likely do not contribute to L3 regeneration.

It has been reported that INCs have the ability to form intercalary neuromasts during late larval development in zebrafish [16, 25, 26]. Here, these cells respond shortly after damage by sealing the gap left by a localized injury. To determine if the INCs are in fact responsible for regeneration of the L3 neuromast, we electroablated L3 in a double transgenic line that labels INCs with GFP and all lateral line cells with an RFP tag: *tg*(*cxcr4b:mCherry;et20:GFP*). Time-lapse imaging of the damaged site after ablation revealed movement of GFP^+^ cells on both sides of the gap that resulted in their reconnection (Additional file 5). In no instance did we observe GFP^−^ cells migrating into the injury zone (*n* = 15). This suggests that cells with INC identity are the only cells that populate the injury site after electroablation.

**Fig. 2:**
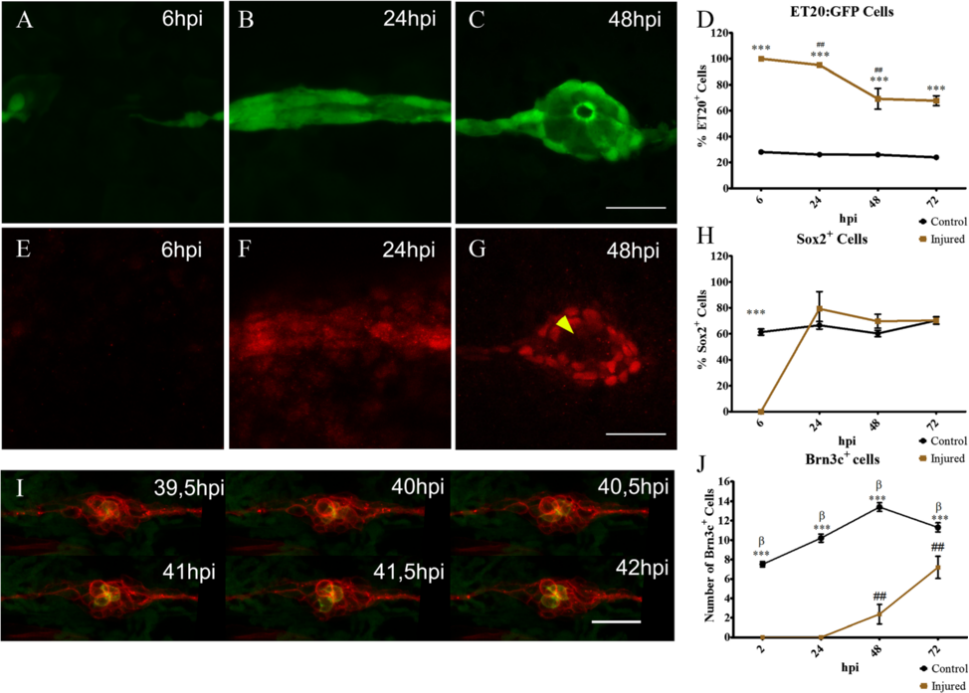
Neuromast regeneration depends on interneuromastic cell accumulation. The L3 neuromasts of 3 days post fertilization *tg*(*et20:GFP*) larvae were electroablated or left uninjured as controls, and fixed at different time points after damage (hours post injury, *hpi*). **a**–**c** Detection of ET20:GFP-labeled cells after electroablation. **d** Quantification of GFP-labeled cells at the L3 position (*n* = 10). Initially, in electroablated fish, all accumulating cells expressed GFP but the percentage of GFP versus total cells diminished significantly between 24 and 48 hpi (## *p* < 0.01). At all stages after injury, L3 neuromasts of electroablated larvae had a much higher proportion of ET20:GFP cells in comparison with control larvae (***p < 0.001). **e**–**h** Immunodetection and quantification of Sox2-expressing cells (*n* = 10). At 6 hpi, few, if any, Sox2-expressing cells were seen in the injury zone but, after 24 hpi, the number of Sox2-expressing cells was approximately the same as in controls (**h**) (***p < 0.001) . Note the loss of Sox2 expression in the most centrally located cells at 48 hpi (**g**, *yellow arrowhead*). **i** Images extracted from a time-lapse sequence of a double *tg*(*cxcr4b:mCherry;brn3c:GFP*) electroablated larva. The sequence reveals the progressive appearance of GFP expression in centrally located hair cells. **j** In vivo quantification of the number of hair cells in control and injured larvae that regenerated their neuromasts at 2, 24, 48, and 72 hpi (*n* = 15); ^##^ and β indicate statistical differences within the same group, control or injured, comparing neighboring values (β *p* < 0.001, ## *p* < 0.01), while asterisks reflect statistical difference between control and injured at the same time points (***p < 0.001). Note that the ET20:GFP and Sox2 expression data corresponding to 6 and 24 hpi (shown in **d** and **h**, respectively), come from a mix of larvae committed and not committed to regenerate. This is because the samples had to be fixed at stages in which we could not distinguish between the outcomes. Scale bar **a**–**g**, i: 50 μm. Further details on replicates are provided in “Quantifications and statistical analysis” in the “Methods” section

We also fixed control and injured larvae at different times points after electroablation (2 or 6, 24, 48, and 72 hpi) with the aim of describing the temporal appearance of the different cell types of the neuromast until the end of the regeneration process. We used the transgenic lines *tg*(*et20:GPF*) for labeling INCs and mantle cells, *tg*(*brn3c:Gap43-GFP*^*+*^) for labeling hair cells, and an anti-Sox2 antibody for detecting progenitor cells [33]. We first confirmed that Sox2 protein is in fact expressed in neuromast progenitor cells and not in INCs. This was indeed the case at all ages analyzed (4, 5, and 6 dpf), shown by the fact that INCs and mantle cells were labeled with GFP in *tg*(*et20:GFP*) transgenic fish whereas only neuromast progenitors were immunostained with anti-Sox2 antibody in the same animals (Additional file 6). As shown in Fig. 2, at 6 hpi the electroablation gap remained devoid of any cells (Fig. 2a, e) but by 24 hpi, INCs (GFP^+^Sox2^−^) from both sides of the gap had connected. At this stage, all the fish examined had a newly established line of INCs (*n* = 40) and some (~29 %) also displayed an accumulation of GFP^+^ cells (Fig. 2b). In the cases where INCs accumulated, some cells began to express the Sox2 progenitor marker (Fig. 2h). At this time point (24 hpi), none of these protoneuromasts had developed hair cells (Brn3c:Gap43-GFP^+^). However, by 48 hpi, 29.2 ± 2.2 % (*n* = 100) of injured larvae exhibited regeneration of the organ (presence of at least two hair cells in the neuromast); this percentage doubled after 72 hpi to 58.2 ± 4.4 % (*n* = 100; see Additional file 1).

In regenerating neuromasts, at 48 hpi, ET20:GFP^+^ cells (also positive for the Sox2 marker) formed the characteristic ring of mantle cells (Fig. 2c) while a group of centrally distributed cells lost the expression of ET20 and expressed only Sox2 (Fig. 2d, h). This was reflected by the recorded decrease in the number of ET20^+^ cells between 24 and 48 hpi (Fig. 2d). Hair cells arose from the central-apical region of the new neuromast, first evident by the loss of ET20:GFP/Sox2 expression and the appearance of the mature hair cell markers (Fig. 2g, yellow arrowhead). After the emergence of hair cells in the regenerating neuromast, we performed time-lapse imaging in a *tg*(*cxcr4b:mCherry;brn3c:gap43-GFP*) larva (Additional file 7; see also Fig. 2i). We quantified hair cell number by in vivo observation of injured larvae during the 3 days following electroablation (Fig. 2j). The number of hair cells increases over time, although there was still a significant difference with respect to controls at at 72 hpi (Fig. 2j). These experiments showed that all of the cell types that compose the mature neuromast develop after the accumulation and posterior differentiation of INCs. However, the lack of appropriate and specific markers to efficiently differentiate accumulating INCs from progenitor or mantle cells prevented us from assigning a direct progenitor role for these cells or determining whether they must first undergo a transition through an intermediate fate.

As described above, we consistently found a proportion of electroablated larvae in which the INC stripe reconnected but that never achieved neuromast regeneration (~40 % of fish). In *tg*(*et20:GFP*) transgenic larvae that did not regenerate the L3 neuromast, INCs continued to express GFP but not the Sox2 protein (see Additional file 6). Thus, achieving INC accumulation and expression of Sox2 can be considered a robust predictor of regenerative success in this context.

### Two INCs are sufficient to form a new neuromast after electroablation

**Fig. 3:**
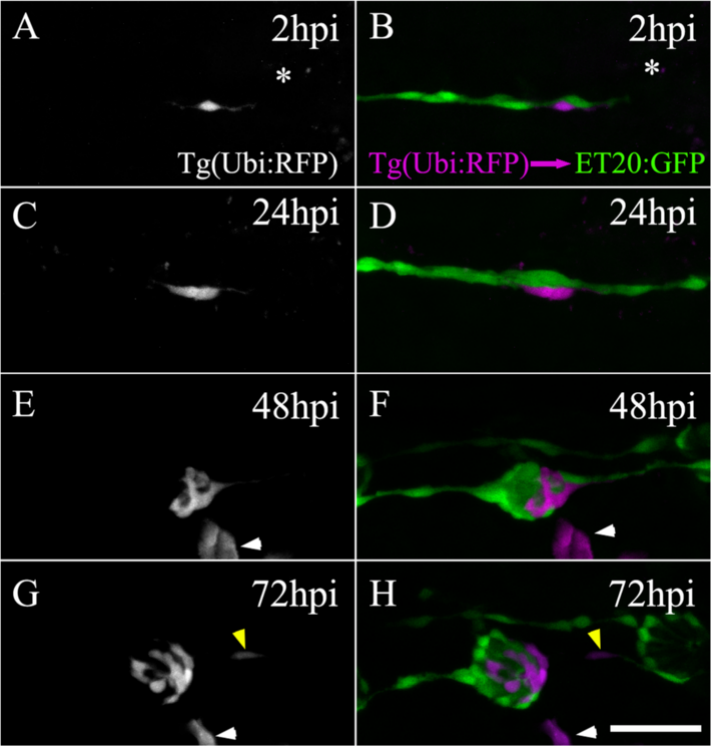
Regenerated neuromasts are chimeric structures derived from two interneuromastic cells (INCs). Transplanting cells from a *tg*(*ubiquitin:RFP*) blastula to a *tg*(*et20:GFP*) blastula occasionally resulted in fish with one or a few labeled INCs. A transplanted larva harboring a single labeled INC near L3 was selected 3 days post fertilization and subjected to electroablation of the L3 neuromast (**a**, **b**). The asterisc indicates the position of the ablated neuromast. The left panels (**a**, **c**, **e**, **g**; only the red fluorescent protein [RFP] channel is shown) show the behavior of the transplanted cell through time. The right panels (**b**, **d**, **f**, **h**) show ET20^+^ cells of host larvae (*green*) and the transplanted cells (pseudocolored in *magenta*). During regeneration, the single transplanted cell divided and its progeny differentiated into different cells types of the mature neuromast. At 72 hpi, a red-labeled cell (here in *magenta*) can be observed among the INCs, suggesting that at least one daughter cell maintained the original identity of the progenitor (**g**, **h**, *yellow arrowhead*). Note that the transplantation experiment randomly generated labeled cells of diverse lineages that did not participate in neuromast regeneration (see for example, **e**–**h**, *white arrowhead*). Scale bar: 50 μm

To elucidate whether INCs are multipotent progenitors able to reconstitute an entire neuromast, including all of its cell types, we generated mosaic animals by transplanting cells (at the high blastula stage, 3 h 20 min) from a *tg*(*ubi:RFP*) donor embryo that ubiquitously expresses a cytoplasmic RFP into a *tg*(*et20:GFP*) host. We screened and selected transplanted larvae 48 hours post fertilization (hpf) that had only one or very few red-labeled INCs (we discarded those larvae that had red mantle cells). In these fish, we were able to follow individual INCs and analyze their behavior during the regeneration process. At 3 dpf, we electroablated the neuromast nearest to the implanted INC in chimeric larvae (Fig. 3a, b, asterisk) and recorded the regeneration process until 72 hpi. As we have shown previously, electroablation generates local damage that is circumscribed to the neuromast without affecting the neighboring INCs. At 24 hpi, INCs accumulated at the injury site. In the example shown, a single red-labeled cell (Fig. 3a) divided into two daughter cells (Fig. 3c), indicating that this accumulation is due to both the migration of INCs into the gap (Additional file 5) and the local proliferation of these cells. From 24 to 48 hpi, these cells continued to increase in number and also began to organize into a rosette-like structure (Fig. 3e, f). At 72 hpi, the number of cells had continued to increase and the newly formed neuromast was apparent (Fig. 3g, h). We also observed labeled daughter cells that remained within the INC population, suggesting self-renewal of the INCs (Fig. 3g, h, yellow arrowhead). Finally, in all cases analyzed (*n* = 10), the regenerated neuromast was composed of a combination of red fluorescent-labeled and ET20:GFP^+^-labeled cells, suggesting that the regenerated sensory organs come from at least two different INCs, most likely the two INCs (rostral and caudal) flanking the ablation gap. In support of this conclusion, we observed that only the most proximal INCs were responsive to neuromast electroablation, given that INCs located in more distal positions remained quiescent and immotile during the regeneration process (Additional file 8, white arrowheads).

In summary, destruction of all cells in a neuromast is followed by convergent migration of at least two INCs into the injury zone. Once there, they proliferate and differentiate, giving rise to all of the cell types of the mature regenerated neuromast.

**Fig. 4:**
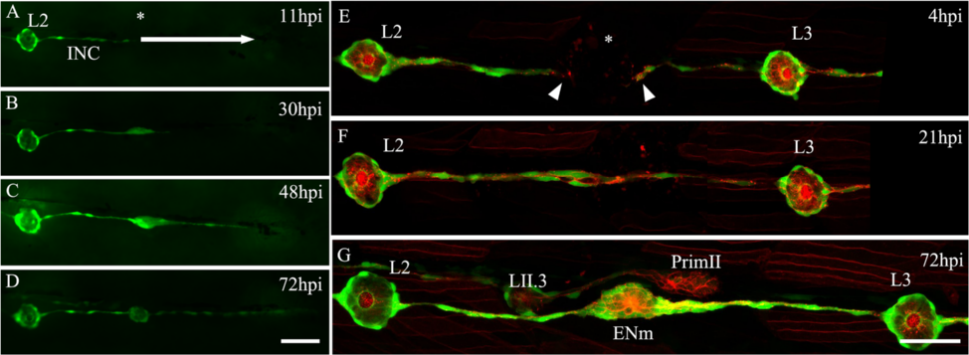
Contribution of interneuromastic cells (INCs) to neuromast regeneration. **a**–**d** Complete elimination of all neuromasts and INCs between L3 and L8 was done 3 days post fertilization in *tg*(*et20:GFP*) larvae by electroablation (*n* = 20). Neuromasts were electroablated whereas INCs were ablated by mechanical displacement of the microelectrode through the skin. The *white arrow* in a shows the direction of the movement of the microelectrode. The *asterisk* in a shows the position of the L3 neuromast before electroablation. After injury, the behavior of INCs located proximal to the gap was examined at 11 hours post injury (*hpi*) (**a**), 30 hpi (**b**), 48 hpi (**c**), and 72 hpi (**d**). Starting at 30 hpi, INCs accumulated at the injury zone and organized to form a new neuromast. They also migrated, beginning at 48 hpi, extending caudally to create a new line of INCs. **e**–**g** An ectopic neuromast can appear de novo after electroablation. **e** The row of INCs between L2 and L3 was interrupted by electroablation at the position of the *asterisk*; the last remaining INCs are indicated by *arrowheads*. **f** At 21 hpi, INCs started to accumulate, reconnecting the line of cells. **g** At 72 hpi, a neuromast formed between L2 and L3 at a position where there was no preexisting neuromast (labeled *ENm*, ectopic new neuromast). At this stage, the secondary primordium (*PrimII*) was migrating close to L3 and had deposited secondary neuromast LII.3. Further details on replicates are provided in “Quantifications and statistical analysis” in the “Methods” section. Scale bar: 50 μm

We next asked whether INCs located only to one side of the injury zone could be responsible not only for neuromast regeneration, but also for the entire lateral line system after more severe damage, as occurs, for example, after tail fin amputation. To examine this question, we electroablated the L3–L8 neuromasts of a 3 dpf *tg*(*et20:GFP*) larvae and mechanically removed all of the INCs posterior to L3. The remaining INCs located near the L3 position migrated caudally (Fig. 4a–d; Additional file 9). These cells started to proliferate and accumulated in the region where L3 was located. At this position, the INCs reorganized into a new neuromast, as we previously described. Then, more distally located INCs that did not belong to the prospective neuromast continued to migrate caudally, iterating the process and reconstituting the canonical row of INCs and neuromasts at the myoseptum (*n* = 20). We never observed the formation of a primordium (collective migration of cells) during this type of lateral line regeneration. We conclude that INCs are progenitor cells that have the capacity, on their own, to restore the entire lateral line system after severe damage. Intriguingly, the progeny of these cells were able to correctly position the new sensory organs to maintain appropriate spacing between them.

We also sought to know if the appearance of a new sensory organ was restricted to the previous location of the damaged neuromast or whether the interruption of the row of INCs by the local application of current at any point could elicit neuromast reconstitution. To investigate this, we electroablated midway between the L2 and L3 neuromasts: 48 hpi we observed the formation of a new neuromast at the ablation point in 28 ± 2.45 % of injured larvae, and at 72 hpi in 56.75 ± 10.05 %, indistinguishable from neuromast ablations (Fig. 4e–g). This result shows that generating a discontinuity in the PLL (including the PLLn, SCs, and INCs) is sufficient to induce the formation of a new neuromast.

### SCs are key regulators of the neuromast regeneration process

As we have previously shown, 60 % of the electroablated larvae regenerate the L3 neuromast after 72 hpi by local activation and differentiation of INCs. However, we were curious why the remaining 40 % of the larvae failed to regenerate a neuromast even though the INCs became activated and migrated to seal the gap between them (Additional file 2). Based on previous knowledge on the development of this sensory system [16, 25, 26], we hypothesized that SCs might be responsible for the control of INC behavior during neuromast regeneration.

**Fig. 5:**
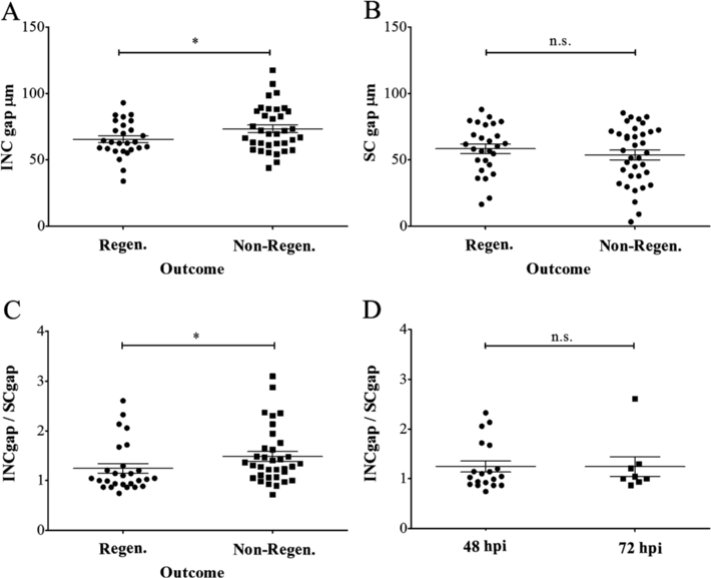
Neuromast regeneration success is inversely correlated with the size of the interneuromastic cells (*INC*) gap generated by electroablation. The L3 neuromast of *tg*(*cxcr4b:mCherry;foxd3:GFP*) larvae was electroablated. At 2 hours post injury (*hpi*), we individually injured larvae and measured the length of the gap between remaining INCs (labeled in *red*) and Schwann cells (*SCs*, labeled in *green*). At 72 hpi, we scored the regeneration of the L3 neuromast and compared the two outcomes (regeneration or no regeneration) after measuring the average gap size in each group. As is shown in **a**, larvae that could not regenerate the L3 neuromast presented a larger gap between INCs compared to those that regenerated (*n* = 64). We did the same comparison examining the size of the SC gap and found no effect in this case (**b**; *n* = 64). Calculating the ratio between INC and SC gap size again produced a significant difference when regenerating versus non-regenerating outcomes were compared (**c**; *n* = 64). Further, there was no difference in the ratio of the INC/SC gap between larvae that regenerated at 48 hpi versus those that regenerated at 72 hpi (**d**; *n* = 27). * *p* < 0.05; *n.s.* not significant. Further details on replicates are provided in “Quantifications and statistical analysis” in the “Methods” section

As described above, both INCs and SCs seal the gap created by electroablation at approximately the same time (24 hpi). To evaluate whether the ability of INCs to regenerate a neuromast depends on their reconnection and local activation before SCs seal the gap, we decided to measure the distance between surviving cells after electroablation in *tg*(*cxcr4b:mCherry;foxd3:GFP*) fish; in this double transgenic line both INCs and SCs are labeled [36, 49]. We correlated the size of the gap created between INCs and between SCs at 2 hpi with the regenerative outcome of injured larvae after 72 hpi. As shown in Fig. 5, we observed that regeneration success was independent of the size of the SC gap. However, the size of the INC gap or, rather, the INC/SC gap size ratio had a significant impact on the regenerative capacity. Fish that failed to regenerate exhibited a higher ratio compared to fish that regenerated the neuromast (Fig. 5c). This suggests that SCs could be interacting with INCs during the early steps of the regeneration process.

**Fig. 6:**
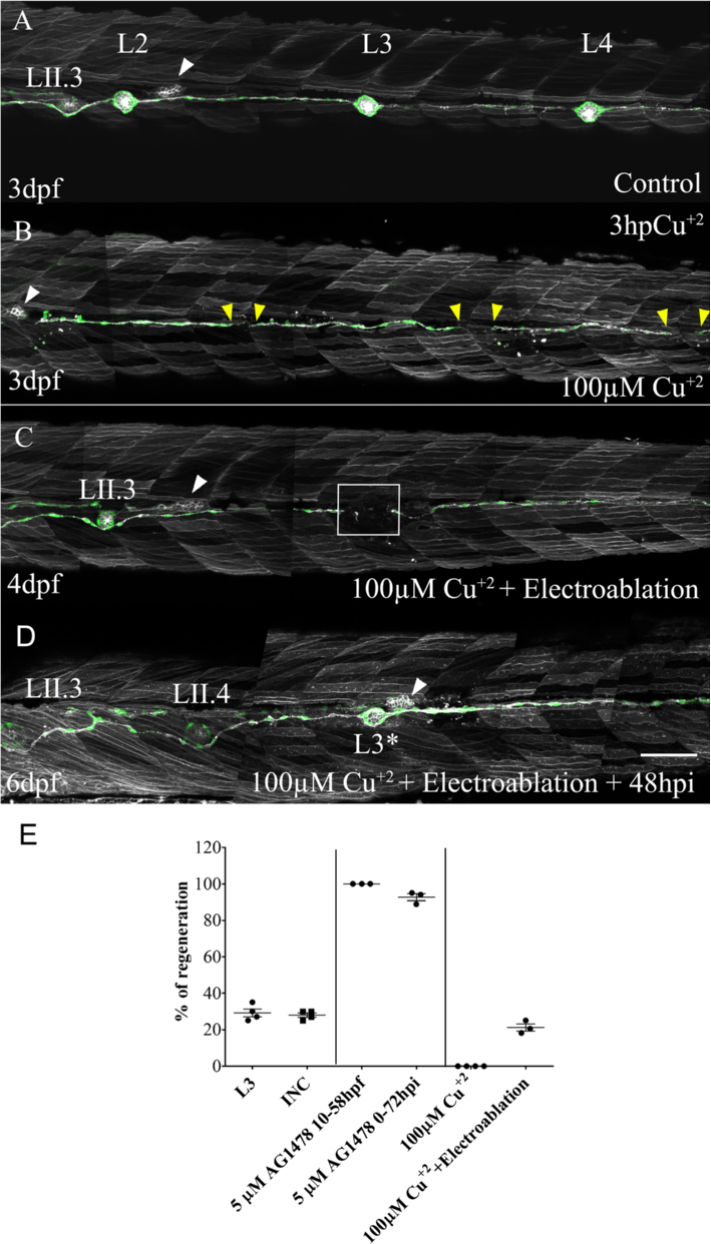
Damage to Schwann cells is required for neuromast regeneration. *tg*(*cxcr4b:mCherry;et20:GFP*) larvae 3 days post fertilization (*dpf*) were treated with 100 μM CuSO_4_ for 2 h to ablate all neuromasts without affecting Schwann cells. **a** A control (uninjured) larva showing the region between L2 and L4. The secondary primordium (PrimII) is seen migrating (*white arrowhead* in all images). **b** Three hours after copper treatment, all neuromasts of the lateral line system had been chemically ablated (gaps demarcated by *yellow arrowheads*). **c** At 24 hours post treatment (4 dpf), interneuromastic cells (INCs) had filled the gaps but no neuromast regeneration occurred. At this time, electroablation was carried out at the approximate position where L3 was (*white box*). **d** A new L3 neuromast formed only where electroablation took place at 48 hours post injury *(hpi*; at 6 dpf). Neither intervention (copper treatment or electroablation) impaired the migration of PrimII (*white arrowhead*) and deposition of secondary neuromasts (LII.3). The *asterisk* indicates the site of injury. **e** The graph shows the percentage of injured larvae that regenerated a neuromast after the different treatments: L3 neuromast electroablation (L3; *n* = 100); electroablation of INCs between the L2 and L3 neuromasts (INC; *n* = 100); L3 neuromast electroablation in larvae treated with 5 μM of AG1478 from 10 hpf until 58 hpf (5 μM AG1478 10–58 hpf; *n* = 75); L3 electroablation in larvae treated with 5 μM of AG1478 from 0 hpi until 72 hpi (5 μM AG1478 0–72 hpi; *n* = 75); 100 μM copper treatment (100 μM CuSO_4,_ *n* = 100); or copper treatment combined with electroablation of L3 (100 μM CuSO4 + Electroablation; *n* = 60). Scale bar a**–**d: 100 μm. Further details on replicates are provided in “Quantifications and statistical analysis” in the “Methods” section

To test the role of SCs in the regenerative capacity of neuromasts, we took advantage of a pharmacological ablation tool that is specific for this cell type. We treated zebrafish embryos with 5 μM of the drug AG1478 from 10 hpf until 58 hpf. This treatment, which blocks ErbB signaling, completely inhibits SC migration along the lateral line nerve during early development [38, 44] without affecting the development of other cellular components of the system. At 3 dpf, after confirming that no SCs were present in the myoseptum, we ablated the L3 neuromast. As shown in Fig. 6e, 100 % of drug-treated larvae showed neuromast regeneration after 48 hpi, compared to only 29 % of the control-injured larvae.

Next, we incubated zebrafish larvae with 5 μM of AG1478 from 2 hours before injury (hbi) until 72 hpi. Treatment at this time does not interfere with early SC migration (when these cells migrate caudally together with the growing PLLn) but impairs their ability to continue to differentiate into mature (myelinating) cells once the drug is added to the medium [44]. In this case, we observed that 92.67 ± 3.21 % (*n* = 75) of injured larvae showed neuromast regeneration after 48 hpi. These results strongly suggest that the presence of differentiated SCs impairs regeneration due to local inhibition of INCs after damage.

In order to effectively test this hypothesis, we decided to damage lateral line neuromasts without affecting the underlying SCs. To this aim, we treated 3 dpf *tg*(*cxcr4b:mCherry;et20:GFP*) larvae with 100 μM CuSO_4_ for 2 h. This treatment leads to complete neuromast loss [13, 31], which was revealed by a discontinuity of the INC line in each of the locations where neuromasts were located (Fig. 6b, yellow arrowhead). Importantly, in this context, neither SCs nor INCs were affected (Additional file 10). After 96 hpi, copper-treated larvae failed to regenerate damaged neuromasts (Fig. 6e; *n* = 100). Surprisingly, when we then electroablated these fish at a point within the reconnected INC row 24 hours post CuSO_4_ treatment (hpt), a new neuromast appeared only where electroablation was done (Fig. 6c, white square). The percentage of larvae that regenerated a neuromast after 48 hpi was 21.21 ± 3.47 % (*n* = 60), similar to that observed in our first electroablation experiment (Fig. 6c–e).

Our results show that the interaction between SCs and INCs is key to control the balance between neuromast formation (regeneration) and replacement with INCs (repair) after damage to the lateral line. The appearance of a new neuromast depends of the temporal and spatial interaction between the two cell types. Finally, the results obtained from AG1478-treated larvae suggests that the interaction between the two cell types in a regeneration context is dependent on the ErbB signaling pathway, as has previously been shown during development of the lateral line system [25].

### Lateral line and single neuromast regeneration in adult zebrafish

**Fig. 7:**
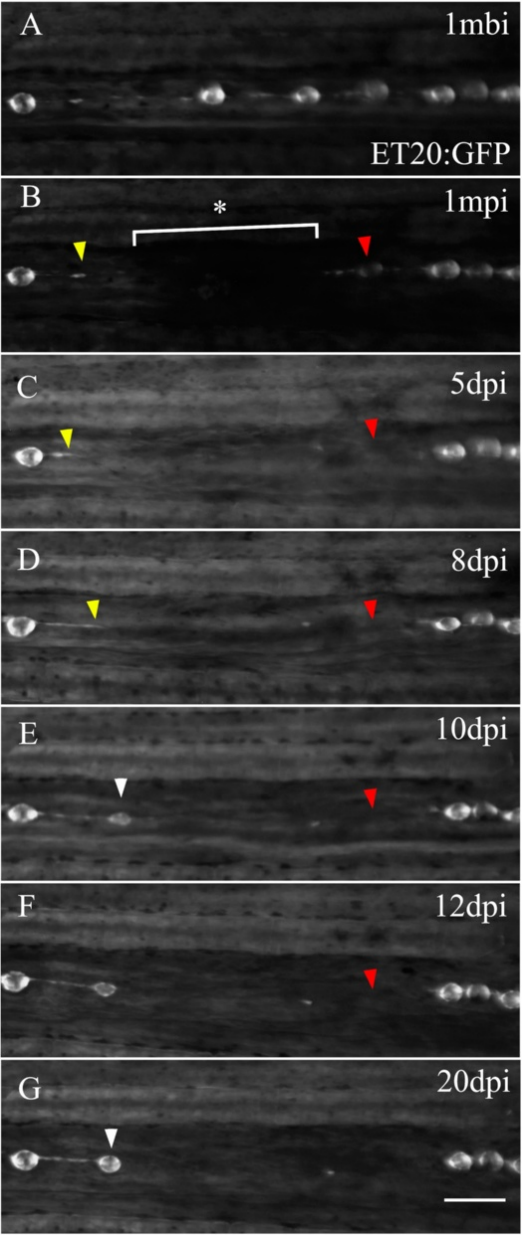
Adult caudal lateral line regeneration after electroablation. A 6-month-old *tg*(*et20:GFP*) fish was electroablated in the caudal fin lateral line by applying two pulses of 2 s duration and 25 μA current intensity (*n* = 60, three independent experiments). Images of the injury were taken beginning from 1-minute post injury (1 *mpi*) to 20 days post injury (*dpi*). The image in **a** was taken at 1 mbi to show the original position of the neuromasts. The *asterisk* and *bracket* in **b** show the extent of the damage. **c**, **d** The *yellow arrowhead* points to the rostral most interneuromastic cells located immediately proximal to the damage. The *red arrowheads* in each panel show the disappearance of the nearest neuromast located caudal to the injury. At 10 dpi, ET20^+^ cells accumulated (**e**, *white arrowhead*) and at 20 dpi had matured into a neuromast (**g**, *white arrowhead*). The position where regenerated neuromasts appeared did not recapitulate the original distribution of neuromasts before injury. Scale bar **a**–**g**: 100 μm

Finally, we wished to know whether the cellular mechanisms used in the larval stage to regenerate the neuromast are maintained in adult fish, where SCs (and all other components of the system) are fully mature. To solve this question, we electroablated an area of approximately 300 μm that usually spans two or more neuromasts in the caudal fin lateral line of a *tg*(*et20:GFP*) adult zebrafish (6 months of age) (Fig. 7). We monitored the regeneration process daily during the following 20 dpi (Fig. 7). We observed the appearance of a regenerated neuromast in the ablated region in 18.33 ± 2.08 % (*n* = 60) of the cases. As seen before in larvae where we ablated all INCs and neuromasts caudal to L3, adult tail fin INCs migrated into the electroablation gap. However, during the time we observed the ablated animals, accumulation of cells to form a new neuromast occurred exclusively on the rostral side of the gap. INCs continued to migrate caudally (Fig. 7b-d, yellow arrowheads) but we did not observe additional neuromasts forming at more posterior positions. On the caudal side of the gap, the last remaining neuromast became gradually disorganized and disappeared 12 dpi (Fig. 7c–f, red arrowheads). This result suggests that the mechanisms employed at larval stages for neuromast regeneration are conserved in adulthood, although the regenerative capacity of neuromasts ablated in this fashion occurs in a lower percentage of individuals.

## Discussion

Regeneration in animals provides functional recovery of damaged tissues and organs after injury or disease and, thus, provides an increased opportunity to survive. For this reason, the mechanisms implicated in tissue regeneration have been extensively studied during the last decades. Regeneration requires progenitor or stem cells to restore a functional organ that usually contains many cell types. Signals that activate these progenitors must be induced after injury to stimulate the stem cells to proliferate and their daughters to differentiate. However, it is equally important for stem cells to remain quiescent when there is no such need. Thus, it is likely that the niche that harbors the stem cells must normally contain inhibitory signals maintaining stasis and multipotency. Discovering such inhibitory mechanisms may be equally as important as searching for the factors that drive activation of stem cells in the context of regeneration.

Here, we have introduced a novel organ regeneration paradigm in zebrafish, an organism in which the regenerative potential of most tissues is high throughout life. Many studies have used the lateral line as a convenient model system for examining the recovery of sensory hair cells and neurons after damage. This is, therefore, an excellent model for acoustic trauma and drug toxicity-induced hearing loss, two of the major causes of permanent deafness in humans. However, physical damage to tissues can also involve the indiscriminate loss of many cell types, which requires the participation of multipotent progenitors to give rise to diverse types of descendant cells. Using electroablation, we showed that we can locally damage a single neuromast eliminating not only the neuromast cells, but also additional lateral line components (PLLn, SCs), the extracellular matrix, skin, and muscle cells. Furthermore, as we described in a recent publication [35], electroablation produces a much more significant inflammatory response in comparison to damage restricted to hair cells [50]. It has been reported that tissue damage induces local release of hydrogen peroxide that can act as a diffusible signal modulating the regenerative response and the recruitment of immune cells to the wound margin [51]. However, this phenomenon clearly does not happen after single cell ablation with, for example, a focused laser.

Despite the intrinsic regenerative capacity of zebrafish larvae, we found that the outcome of this treatment differed among individual fish, as not all could replace the lost neuromast. This unexpected result led us to hypothesize that perhaps the same mechanism that limits the formation of neuromasts from progenitors during development could be operating in this context, at least in a percentage of the treated animals. By following the regeneration process in vivo, we were able to resolve this question by showing that SCs are key modulators of lateral line regeneration.

### SCs and INCs interact during neuromast regeneration

Regeneration assays using the zebrafish lateral line system have been widely used but they have revealed an apparent contradiction. On the one hand, exposure to high concentrations of copper sulfate irreversibly destroys the lateral line neuromasts, precluding regeneration even though there is no effect on surrounding tissues or on INCs [31, 33]. On the other hand, in a tail fin section paradigm, it has been shown that lateral line cells, most likely from the INC population, are able to proliferate and invade the regenerated tail and form new neuromasts [9]. These results leave open questions regarding the cellular mechanisms and conditioning factors involved in the restoration of an entire neuromast after the removal of all of its cells. Thus, as a first step to understand how neuromasts regenerate, it is fundamentally important to visualize how different cell types interact during the regeneration process by following their behavior in vivo.

During the initial stages of lateral line development, growth of the PLLn does not have any role in the deposition of neuromasts by the PrimI, but it is necessary for the migration of SC precursors along the lateral line [26, 49]. As it grows, the PLLn expresses Neuregulin I type III (Nrg1-3). This protein is involved in the migration, proliferation, and differentiation of the SCs by binding to ErbB receptors expressed in SCs [28]. This tripartite relationship between the PLLn, SCs, and PrimI-derived cells is a hallmark of this system. It has been shown that the absence of the nerve leads to the loss of the glia in the PLL [16] and induces the formation of supernumerary neuromasts [16, 25, 26]. As we show here, after neuromast damage, these three elements interact during the regeneration process.

Electroablation of the L3 neuromast eliminates all neuromast cells in the injury zone (complete absence of Brn3c^+^, Sox2^+^, and ET20^+^ cells, which are hair cells, neuromast progenitors, and mantle cells, respectively). Also, the lateral line nerve is interrupted and degenerates from the injury point caudally. The degeneration of the PLLn rapidly induces the loss of Nrg1-3 signaling, which interrupts SC differentiation, as seen in other models [38–44]. We revealed this by showing the temporary loss of myelination (MBP expression) soon after electroablation in SCs caudal to the lesion. Despite this dedifferentiation effect in SCs, we did not detect formation of intercalary or ectopic neuromasts posterior to L3 in electroablated fish. Thus, the signal-inhibiting activation of INCs is either continually produced by the dedifferentiated SCs or it persists (perhaps associated with the extracellular matrix) after denervation. Twenty-four hours after injury, the nerve regenerates and reinnervates the caudal neuromasts while glial cells, located at both sides of the gap, make use of regenerated axons to reconnect, and start to differentiate once again [38].

Reports describing amphibian lateral line regeneration after tail amputation indicate that new lateral line cells can arise from existing neuromasts; for example, mantle cells detach from the neuromast and migrate to form a new lateral line [47]. We examined neighboring neuromasts (L2 and L4) after ablating L3 and found no evidence for changes in cell composition or the number of cells in them. This suggests that progenitor cells residing within surviving neuromasts do not contribute to the regenerative process. Further, we ablated all neuromasts using a high dose of copper sulfate and were able to generate a new neuromast by electroablation (Fig. 6); thus, lateral line neuromasts can form in larvae in the absence of any other neuromast cells, indicating that progenitors must come from INCs, the only cell population remaining in this case.

After damage to the L3 neuromast in larvae, INCs located close to the injury zone migrated into the gap and reconnected the line of cells. We observed that this process involved a bilateral contribution of cells located on both sides of the gap. Further, just two of the convergent INCs were required to form the entire regenerated neuromast with all of its cell types. Therefore, INCs are a multipotent population of cells that are normally quiescent and can become proliferative and give rise to differentiated progeny when an injury occurs.

Notably, in larvae, a functional neuromast formed at approximately the same position of the original neuromast in a matter of 2 days. This is different from what we observed in adults. In the adult tail fin, regeneration of an ablated section of lateral line took over 20 days. During this time, we noticed that the closest neuromast located caudal to the injury became disorganized and disappeared. This did not occur with rostrally located neuromasts. It is known that afferent innervation of adult neuromasts is required for their maintenance [52]. Since we were ablating the nerve, and the regenerative process is slow compared to larval stages, it is possible that adult neuromast cells caudal to the damaged site lost the signaling required for their continued survival. In addition, electroablation of the adult tail produces a potent inflammatory response [35], which could induce tissue remodeling and local destruction of cells, as occurs in larvae after copper treatment [50]. In addition to the disappearance of the first neuromast caudal to the injury area, we also failed to observe any contribution of caudal INCs to the injury zone. Further exploration of this phenomenon will be needed in order to clarify the role of innervation and/or inflammation in the regeneration of the adult lateral line.

As a consequence of the unilateral contribution of INCs to PLL regeneration in adult tail neuromasts, the new neuromasts reappeared in locations that were different to the original ones. This also occurred in larvae when we eliminated the entire lateral line from L3 posteriorwards or after electroablating between neuromasts (Fig. 4). These data are consistent with the hypothesis that the ability of INCs to form a neuromast is strictly dependent on the absence of the inhibitory signal produced by SCs, and that this signal is likely stored in the extracellular matrix: in the total absence of glia, neuromasts are “free” to form in any location, as long as the INCs accumulate and differentiate.

### SCs inhibit INC activation via the ErbB signaling pathway

Despite the consistency of the neuromast electroablation process in larvae, we found two distinct outcomes after 3 days of recovery: regeneration of the neuromast (60 % of larvae) or reconnection of the INCs without the formation of a new organ (40 % of cases). We noticed that INCs and SCs mobilized and filled the gap almost simultaneously, indicating that perhaps there was an almost equal chance of regeneration versus repair depending on a small advantage of one cell type versus the other when reconnecting both sides of the gap. Several experiments confirmed the crucial role SCs play in controlling INC fate. First, we showed that when we ablated neuromasts without affecting SCs (by copper sulfate treatment), INCs migrated and reconnected to restore the stripe of cells but regeneration of the neuromasts never occurred; regeneration could be induced in this case only by electroablating (which locally eliminated SCs). Second, when we inhibited SC development by inhibiting ErbB signaling, the neuromast regeneration efficiency rose to 100 % in electroablated larvae. We showed that ErbB signaling is in fact critical for the inhibitory effect because regeneration efficiency could also be increased by inhibiting the pathway during the process (exposing fish from 2 hbi to 72 hpi).

Thus, neuromast regeneration depends on the generation of a spatial niche that is free of inhibition by SCs during a temporal window that is sufficient to induce INC migration and proliferation; this is likely the same process that occurs during formation of an intercalary neuromast in normal development of the lateral line in juvenile fish.

### INCs are multipotent progenitors

**Fig. 8:**
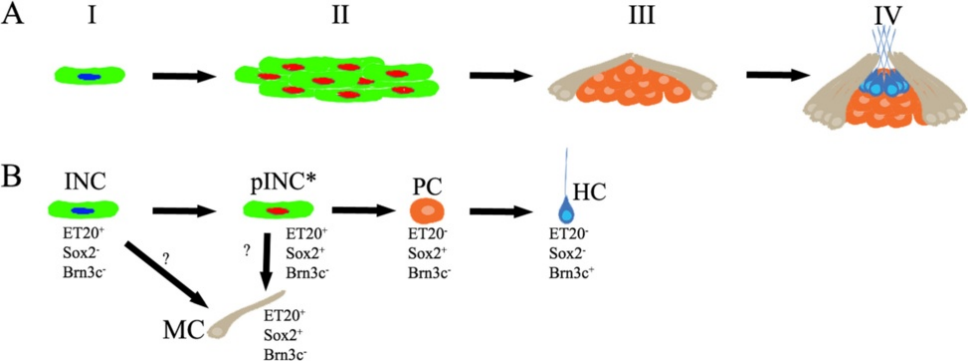
Schematic representation of neuromast regeneration from interneuromastic cells (*INCs*). **a** A schematic representation of the four stages identified here during regeneration of a whole neuromast. I, after neuromast ablation, a remaining INC that has multipotent progenitor properties (*green with blue nucleus*, ET20:GFP^+^) becomes activated; II, the INC divides, and daughter cells accumulate where the original neuromast was and acquire expression of Sox2 (*green with red nuclei*; ET20:GFP^+^, Sox2^+^); III, mantle cells (*brown*) differentiate at the periphery of the cell cluster while central cells lose GFP expression (ET20:GFP^−^, Sox2^+^), becoming neuromast progenitor cells; IV, neuromast regeneration is achieved when hair cells (*blue*) differentiate. **b** Differentiation pathway of INCs during neuromast regeneration. It is unclear from our study whether INC progeny can differentiate directly into the mantle cells (*MC*) or whether MCs differentiate from a proliferative INC (pINC*) (an ET20^+^Sox2^+^Brn3c^−^ cell). The pINCs accumulate and can differentiate into a neuromast progenitor cell (*PC*), losing the ET20 marker. Finally, the acquisition of Brn3c expression (hair cells, *HC*)

With the tools available to us, we have been able to describe the process INCs follow to regenerate a functional neuromast after the preexisting one has been electroablated. INCs normally express the ET20:GFP marker but, as they become activated to migrate, proliferate, and accumulate at the damage site, they temporarily co-express the Sox2 protein (Fig. 8a). Some of these cells (at the center of the cluster) then lose the ET20:GFP marker, retaining only Sox2 expression, thus becoming neuromast progenitor cells [33]. Importantly, we determined that the loss of ET20:GFP and retention of Sox2 in a central subset of cells is strictly correlated with a regenerative outcome. Those cells that continue to express both markers accumulate at the periphery of the forming neuromast and become mantle cells. However, in the absence of a more precise definition of lineage relationships, we do not know if all cells transition through one of these two conditions and then one subset differentiates into the other, or whether there are two independent lineages arise from INCs that generate both populations (Fig. 8b). Towards the end of the regeneration process, the apical centrally located cells lose the ET20:GFP and Sox2 labels and start to express Brn3c, a hair cell marker indicative of differentiation. This differentiation process likely involves Notch signaling, the pathway responsible for blocking proliferation and the loss of progenitor status [53]. Using genetically mosaic animals, we were able to show that all of the cell types of a regenerated neuromast can derive from a single INC. In all cases examined, we saw that at least two INCs contributed to the regenerated organ; thus, we propose that one INC from each side of the wound is sufficient to give rise to the complete neuromast.

## Conclusions

In this study, we explored the cellular mechanisms that govern neuromast regeneration and how the specialized cellular microenvironment constrains the behavior of progenitor cells responsible for regenerating an organ or tissue. We show that the mechanism involved in the normal development of the lateral line during the formation of intercalary neuromasts is also used during regeneration of neuromasts in larvae and adult zebrafish. INCs are multipotent progenitors that are kept quiescent by ErbB signaling in SCs, but a traumatic event that disrupts this inhibition can trigger regeneration. This simple yet highly effective interrelationship between a stem cell and a regulatory cell can be useful for defining the properties of a niche that can provide the cellular substrate for repeated and permanent tissue regeneration.

## Methods

### Zebrafish husbandry and transgenic lines

Zebrafish (*Danio rerio*) embryos were obtained by natural spawning of the following transgenic strains: *tg*(*neurod:EGFP*)^*nl1*^ [54], *tg*(*brn3c:GAP43-GFP*)^*s356t*^ [55], *tg*(*cxcr4b:mCherry*)^*ump1*^ [36], *tg*(*foxd3:GFP*)^*zf104*^ [49], *tg*(*Ubi:zebrabow:cherry*) [56], and *Et*(*krt4:EGFP*)^*sqet20*^ [herein called *tg*(*et20:GFP*)] [48]. The embryos were staged according to Kimmel et al. [57]. We express larval ages in hours post fertilization (hpf) or days post fertilization (dpf). Fertilized eggs were raised in petri dishes containing E3 medium (5 mM NaCl, 0.17 mM KCl, 0.33 mM CaCl2, 0.3 mM MgSO4, and 0.1 % methylene blue) until 96 hpf. Most of the experiments were carried out in 72 hpf larvae, because at this stage the primary lateral line is completely developed and functional [18]. All procedures complied with national guidelines of the Animal Use Ethics Committee of the University of Chile and the Bioethics Advisory Committee of Fondecyt-Conicyt (the funding agency for this work).

### Single neuromast electroablation

A single neuromast was electroablated at 3 dpf as described by Moya-Díaz *et al.* [35]. Briefly, 3 dpf larvae were anesthetized with 0.01 % tricaine and mounted in rectangular plates sealed with low melting point agarose (1 %) dissolved in E3 medium. Once the agarose was set, the embryos were exposed to an electrical pulse locally applied over a single neuromast using a tungsten electrode (FHC Inc., Bowdoin, ME, USA) connected to a power source under the following conditions: two pulses of 2 s duration and an 8 μA current intensity. After this procedure, the larvae were dismounted and analyzed under a fluorescent microscope. Successfully ablated larvae were selected and maintained in E3 medium.

The same experiment was performed on adult fish. In this case, we ablated lateral line neuromasts located in the tail because of their accessibility. Fish were anesthetized with 0.1 % tricaine dissolved in water. The adults were placed directly on rectangular plates and the ablation was performed under the following conditions: two pulses of 2 s duration and a 25 μA current intensity. Each adult was treated individually and observed in a fluorescent scope every 24 h in order to describe the entire regeneration process.

### Multiple neuromast and INC ablation

The complete ablation of the L3–L8 neuromasts was carried out by electroablation of each neuromast as has been described previously, and the INCs located between them were mechanically removed by sliding a microelectrode through the skin (“scratching”) in double *tg*(*et20:GFP; cxcr4b:mCherry*) larvae. The complete ablation of the INCs and neuromast cells was verified at 3 hpi by complete absence of mCherry^+^ cells and GFP^+^ cells at the myoseptum.

### Transplantation experiments

Donor and host embryos were raised in E3 medium until the high stage (3 h 20 min). At this stage both groups of embryos were dechorionated by incubation with pronase (0.2 mg/mL) and maintained in Holtfreter’s solution (NaCl 59 mM; KCl 0.67 mM; CaCl_2_ 0.9 mM; MgSO_4_ 0.81 mM; NaHCO_3_ 2.38 mM) with penicillin/streptomycin (5000 U/L; 100 mg/L; Sigma, St. Louis, MO, USA). Approximately 10–20 donor cells were aspirated and transplanted into host embryos at the same stage using a micropipette connected to a 1 mL syringe as described previously [58]. Host embryos were screened for the presence of donor cells in the PLL at 48 hpf.

### Drug treatments

We performed conditional inhibition of ErbB signaling using AG1478, a competitive kinase inhibitor that is routinely used in zebrafish to block this signaling pathway [37, 44, 59, 60]. A final concentration of 5 μM AG1478 (Calbiochem, San Diego, CA, USA) in 0.05 % dimethyl sulfoxide (DMSO) was added to the E3 medium containing larvae from 10 hpf until 58 hpf to inhibit SC development. Alternatively, a final concentration of 5 μM of AG1478 in 0.05 % DMSO was added to embryos between 60 hpf and 144 hpf. This treatment allows SC development but blocks ErbB signaling from 12 h before electroablation until the end of the regeneration process.

For copper treatment, CuSO_4_ (Merck, Darmstadt, Germany) was dissolved in E3 medium and 72 hpf larvae were exposed to 100 μM CuSO_4_ for 2 h and subsequently rinsed a minimum of three times in fresh E3 medium.

### Immunohistochemistry

Whole-mount immunohistochemistry was performed using rabbit anti-GFP (1:500, A11122, Invitrogen, Carlsbad, CA, USA), mouse anti-GFP (1:500; MAB3580, Millipore, Billerica, MA, USA), rabbit anti-MBP (1:50; kindly provided by Dr William Talbot), rabbit anti-Sox2 (1:500; AB97959; Abcam, Cambridge, UK), Alexa 488 goat anti-mouse (1:2000; Invitrogen, 11029), Alexa 594 goat anti-rabbit (1:2000; Invitrogen, A31632), Alexa 488 goat anti-rabbit (1:2000; Invitrogen, 11034), and Alexa 594 goat anti-mouse (1:2000; Invitrogen, A11032) following standard procedures. Briefly, larvae were rehydrated from methanol, rinsed in 0.3 % phosphate-buffered saline (PBS)-TritonX-100, permeabilized with proteinase K (40 μg/mL, 40 min for 3 dpf larvae and 70 min for 5–7 dpf larvae), washed, and refixed in 4 % paraformaldehyde, transferred to blocking solution (2 % normal goat serum, 1 % DMSO, and 1 mg/mL bovine serum albumin [BSA] in PBS with 0.1% Tween) for 45 min. Larvae were then incubated overnight at 4 °C with primary antibodies, washed with 0.3 % PBS-TritonX-100, and incubated for 2 h at room temperature with secondary antibodies dissolved in blocking solution (10 % p/v BSA, 2 % v/v goat serum, 1 % v/v DMSO, 0.1 % v/v TritonX-100).

For MBP immunohistochemistry, the larvae were treated with three cycles of cold sodium citrate buffer (10 mM, pH 6) of 10 min each, followed by 1 min with citrate at 90 °C after proteinase K treatment. For Sox2 immunohistochemistry, the larvae were not treated with proteinase K. The percentage of Sox2^+^ or ET20^+^ cells was calculated by the co-localization of markers using confocal z-stack sections of the fish with all nuclei labeled with TO-PRO-3.

### Image acquisition and time-lapse imaging

The PLL was imaged in stable transgenic *tg*(*neurod:GFP*), *tg*(*cxcr4b:mCherry*)^*ump1*^*, tg*(*brn3c:GAP43-GFP*)^*s356t*^, *tg*(*foxd3:GFP*)^*zf104*^, *tg*(*Ubi:zebrabow-M*), and *Et*(*krt4:EGFP*)^*sqet2*0^ [herein designated *tg*(*et20:GFP*)] zebrafish larvae. Embryos were anaesthetized in 0.01 % tricaine and mounted on sealed agarose plates. A Zeiss (Oberkochen, Germany) confocal microscope (LSM 510 meta) was used to image lateral line nerve and SCs, INCs, and neuromast cells with ×10 or ×25 objectives. The assembly of the images to display the entire larvae was done using Adobe (San Jose, CA, USA) Photoshop CS5.

For time-lapse analysis, embryos were imaged at various intervals after electroablation for about 12 h on a confocal microscope with a × 25 water immersion objective (Zeiss LSM 510 meta). Approximately 20 confocal sections of the recommended thickness according to the objective were gathered at each time point into maximum projections and compiled into movies with ImageJ software. Embryos were maintained at 28 °C with a heat stage throughout imaging.

### Quantifications and statistical analysis

The number of cells at different time points during the regeneration process was quantified using the *cell counter* plugin of ImageJ. We used ANOVA for treatment comparison or an equivalent nonparametric method (Kruskal-Wallis), depending on the structure of the data. Additionally we used a two-way ANOVA when the measured parameter depended on two factors. The significance level was *p* < 0.05 for all treatments. All data analysis was performed using Prism 5.0b (GraphPad Prism Software, Inc., La Jolla, CA, USA).

The number of animals used varied in each experiment. We usually used 10–25 animals (*n*) for each experiment and data were collected from three or four experiments (*N*) to generate a data point. Specifically, the following number of animals were used: In Fig. 2d, h, each point is an average of three experiments (*N* = 3), each consisting of 10 larvae (*n* = 10) whereas in 2J *n* = 15 larvae; in Fig. 5a–c, *n* = 64 and 5d, *n* = 27 (considering a total of animals in each pairwise comparison); in Fig. 6e, each data point is an average of findings in 25 larvae (*n* = 25), except for the experiment “100 μM Cu + 2 + Electroablation” in which *n* = 20, and we carried out three or four replicates (*N* = 3 or 4); for Additional file 1, *n* = 25 and *N* = 4. For other figures, *n* is indicated in the legend; data analysis was performed on animal replicates.

## Additional files

Additional file 1: The non-regenerative outcome of neuromast electroablation results in restoration of a line of INCs at the site of injury. The L3 neuromast of transgenic *tg*(*cxcr4b:mCherry; brn3c:Gap43-GFP*) larvae was electroablated and the outcome was scored as regeneration if at least two Bn3c^+^ cells (hair cells) appeared in the injury zone after the indicated time (*n* = 100). (A) Quantification of the result; the data are expressed as the percentage of larvae that regenerated the L3 neuromast at different time points after injury. The first regenerated neuromasts can be detected starting from 48 hpi in 29.2 ± 2.2 % of cases. This percentage increases significantly and reaches 58.2 ± 4.4 % after 72 hpi. From 72 to 96 hpi, the percentage of larvae that regenerate neuromasts remains unchanged. (B–D) A 3 dpf *tg*(*et20:GFP*) fish was transplanted at the blastula stage with cells from a *tg*(*ubiquitin:RFP*) donor fish (transplanted cells pseudocolored in magenta) and its L3 neuromast was electroablated (asterisk in B). In 40 % of cases, the INCs migrate to the injury site, proliferate, but fail to accumulate and organize into a mature neuromast. Yellow arrowheads show the location of the different transplanted cells through time. Scale bar B–D: 50 μm. Further details on replicates are provided in “Quantifications and statistical analysis.” (TIF 4880 kb) Additional file 2: Behavior of the lateral line nerve after neuromast electroablation. *g*(*neurod:GFP; cxcr4b:mCherry*) larvae that express mCherry in all cells types of the lateral line and GFP in the lateral line nerve were electroablated at 3 dpf in the L3 neuromast (asterisk); images show only the GFP channel. (A) At 3 hpi, degenerating fragments of denervated axons are still present at the myoseptum (white arrowheads). (B) At 9 hpi, nerve fragments have been cleared and axons sprouting from the nerve stump begin to extend and explore the injured region (yellow arrowhead). (C) From 9 to 27 hpi, the nerve regrows caudally (yellow arrow). The asterisks indicates the injury site and the yellow arrowhead shows axons innervating this region. Also, the regrowing axons innervate more caudal neuromasts (white arrow). (D–F) Higher magnification of the injury zone at 3 hpi (D), 9 hpi (E), and 27 hpi (F). Scale bar: 100 μm. (TIF 9198 kb) Additional file 3: Temporary loss of MBP expression in caudally located SCs after L3 electroablation. *tg*(*foxd3:GFP; brn3c:GFP*) larvae 3 dpf were injured at the L3 neuromast and fixed at different time points. Expression of Brn3c:GFP was used as an indicator to locate the L3 neuromast. Fixed control and injured larvae were processed by immunohistochemistry to analyze MBP and GFP expression. (A, B) White arrowheads show MBP signal in 4 dpf control larvae and 24 hpi in injured larvae (only rostral to L3). The yellow arrowhead in B shows the loss of MBP expression in the caudal zone with respect to the injury site. (C–N) Double transgenic larvae *tg*(*foxd3:GFP; brn3c:GFP*) were processed in order to describe, by immunodetection, the time course of MBP expression in the vicinity of L3 in control larvae (C–H) vs injured larvae (I–N). MBP expression by itself is shown in monocolor (C, E, G, I, K, M), while MBP expression (red) merged with the FoxD3 expression (green) is shown in color (D, F, H, J, L, N). In control larvae, there is continuous expression of MBP from 3 to 5 dpf. The arrowhead in C shows the localization of the L3 neuromast. In injured larvae, the expression of MBP is fragmented after 3 hpi (white arrowhead in I), disappears after 24 hpi caudal to L3 (yellow arrowhead in K), and reappears after 48 hpi (M). Note that, despite the transient loss of the differentiation marker, SCs revealed by the presence of GFP are present throughout the regeneration process (J, L, and N). The asterisk in I and J indicates the site where injury was made. Scale bar A, B: 200 μm, C–N: 50 μm. (TIF 6575 kb) Additional file 4: Electroablation of a neuromast does not affect the cellular composition of neighboring neuromasts. Triple transgenic *tg*(*cxcr4b:mCherry; brn3c:Gap43-GFP; et20:GFP*) larvae were electroablated 3 dpf in order to injure the L3 neuromast. Control (black lines) and injured larvae (brown lines) were then processed to determine the cellular composition of neighboring neuromasts located rostral and caudal to the injury zone (L2 and L4, respectively). (A, B) In vivo quantification of Brn3c^+^ hair cells in the L2 (A) and L4 (B) neuromasts (*n* = 13) at 2, 24, 48, and 72 hpi in control (black line) and injured larvae (brown line). In all subsequent experiments, quantification times are 6, 24, 48 and 72 hpi. (C, D) Quantification of progenitor cells (Sox2^+^ET20^–^) in the L2 and L4 neuromasts (*n* = 12). (E, F) Double Sox2^+^ and ET20^+^ cells were detected by double immunostaining (*n* = 12). (G, H) The total cell number in neuromasts was quantified by TO-PRO-3 staining. Only cells located within the typical ring structure of the neuromast were counted (*n* = 12). There are no changes in L2 or L4 neuromast composition after L3 damage. Further details on replicates are provided in “Quantifications and statistical analysis.” (TIF 159 kb) Additional file 5: (Movie 1) Reconnection of the INCs by bilateral convergent migration. Time lapse of the injury zone of a *tg*(*cxc4b:mCherry; et20:GFP*) from 9 hpi to 19 hpi; images were captured every 25 min. It is possible to observe muscle degeneration after injury by following Cxcr4b^+^ Et20^–^ cells. The INCs (Cxcr4b^+^ Et20^+^ cells) extend process from both sides towards the mid point until contacting each other. (AVI 1727 kb) Additional file 6: INCs do not express Sox2 protein. *tg(et20:GFP)* transgenic larvae at 4, 5, and 6 dpf were fixed and immunolabeled with the anti-Sox2 antibody, revealed with red fluorescence. The top row (A, D, G) shows both GFP signal and Sox2 immunostain; middle row (B, E, H), Sox 2 expression; and bottom row (C, F, I), GFP label. Transgene-driven GFP expression is seen in INCs and in mantle cells of neuromasts. Sox2 expression is only detected in neuromasts, in progenitor cells that underlie hair cells, and in mantle cells (see [33]). At these stages, both primary (L1, L2, etc.) and secondary (LII.1, LII.2, etc.) neuromasts are present and the advancing PrimII (also expressing Sox2) can be seen. (TIF 5802 kb) Additional file 7: (Movie 2): Accumulated INCs can differentiate into mature hair cells. The differentiation process was followed by time-lapse imaging of the injury zone of a *tg*(*cxc4b:mCherry; brn3c:GAP43-GFP*) from 30 hpi to 42 hpi every 30 min. Hair cells express GFP (green). (AVI 990 kb) Additional file 8: INCs respond to damage and behave like multipotent progenitor cells. Blastula cells from Tg(ubiquitin:RFP) embryos transplanted into *tg*(*et20:GFP*) embryos result in chimeric larvae with a few labeled INCs (magenta, labeled with arrowheads) that can be followed through time in vivo. (A) At 3 dpf, a chimeric larva was electroablated in the L2 neuromast; the asterisk shows the injury site. A proximal (yellow arrowhead) and more distal (red and white arrowheads) labeled INCs can be observed. (B) After 24 hpi, the INC proximal to the injury zone has proliferated and daughter cells begin to accumulate (yellow arrowheads) whereas the nearest neighboring INC seen in A divides once but the daughter cells do not move from their position (red arrowheads). The farthest labeled INCs (white arrowheads) do not divide. (C) After 72 hpi, a regenerated L2 neuromast appears in the position where the injury was made. With the exception of two cells, the entire regenerated L2 neuromast is derived from the single labeled INC cell (yellow arrowhead in A). The distal INC has divided once more but does not contribute to the regenerated neuromast (red arrowheads). From 24 hpi to 72 hpi (which corresponds to 4–6 dpf), the PrimII travels through this region and deposits the secondary neuromasts LII.1 and LII.2. Scale bar: 50 μm. (TIF 1069 kb) Additional file 9: (Movie 3) Unilateral contribution of INCs can result in neuromast regeneration. Electroablation of all cells between the L3 and L8 neuromasts in a 3 dpf tg(ET20:GFP) was carried out by electroablating and mechanically sliding the microelectrode through the skin, removing all of the INCs posterior to L3. The caudal migration of INCs and their accumulation to form a new neuromast was followed by time-lapse imaging from 4 hpi to 34.5 hpi (images captured every 30 min). Note the beginning of the INC accumulation process towards the end of the sequence. (AVI 806 kb) Additional file 10: Copper sulfate treatment does not affect SCs or INCs. (A, B) 72 hpf *tg*(*foxd3:GFP*) transgenic larvae (SCs labeled with GFP) were treated for 2 h with 100 μM CuSO_4_ and imaged 1, 24, and 48 hpt; sibling control fish were left untreated. (A) An arbitrary area of the trunk above the anus was defined (encompassing the width of about three somites) and the “Measure Tool” of ImageJ was used to assign arbitrary units for mean green fluorescence intensity. (B) The graph shows data for control (Co) and copper-treated (CuSO_4_) fish at the three selected times. While mean values are significantly different between the developmental stages, control fish vs CuSO_4_-treated fish (control 1, 24, 48 h *n* =12, 14, 11 respectively and 1, 24, 48 hpt *n* =13, 17, 11 respectively) at equivalent ages were not significantly different (ns) (*P* < 0.05). (C) Double transgenic *tg*(*cxcr4:mCherry; et20:GFP*) larvae at 3 dpf were treated for 2 h with 100 μM CuSO_4_ and imaged 48 and 72 hpt. Two larvae are shown at both time points. Note that only secondary lateral line neuromasts (LII) are seen because the copper treatment at day 3 permanently ablates all primary neuromasts. In the bottom three rows, sibling fish were treated in the same way but, after the copper solution was washed out, 5 μM AG1478 was added to the medium and the fish were imaged 48 or 72 hours later; three larvae are shown at both time points. Note that supernumerary neuromasts appear in these animals (compare with two larvae in top half), indicating that INCs are not affected by copper treatment and retain their progenitor potential. (PDF 1994 kb)

## Abbreviations

BSA: bovine serum albumin
DMSO: dimethyl sulfoxide
dpf: days post fertilization
dpi: days post injury
GFP: green fluorescent protein
hpf: hours post fertilization
hbi: hours before injury
hpi: hours post injury
hpt: hours post treatment
INCs: interneuromastic cells
MBP: myelin basic protein
mCherry: monomeric Cherry fluorescent protein
PBS: phosphate-buffered saline
PLL: posterior lateral line
PLLn: posterior lateral line nerve
PrimI: migratory primordium
PrimII: secondary primordium
RFP: red fluorescent protein
SCs: Schwann cells
