# Supplementary figures and images for: Mechanosensory organ regeneration in zebrafish depends on a population of multipotent progenitor cells kept latent by Schwann cells
Source: BMC Biol. 2016 Apr 7;14:27. doi: 10.1186/s12915-016-0249-2 (PMC4823859; doi:10.1186/s12915-016-0249-2)

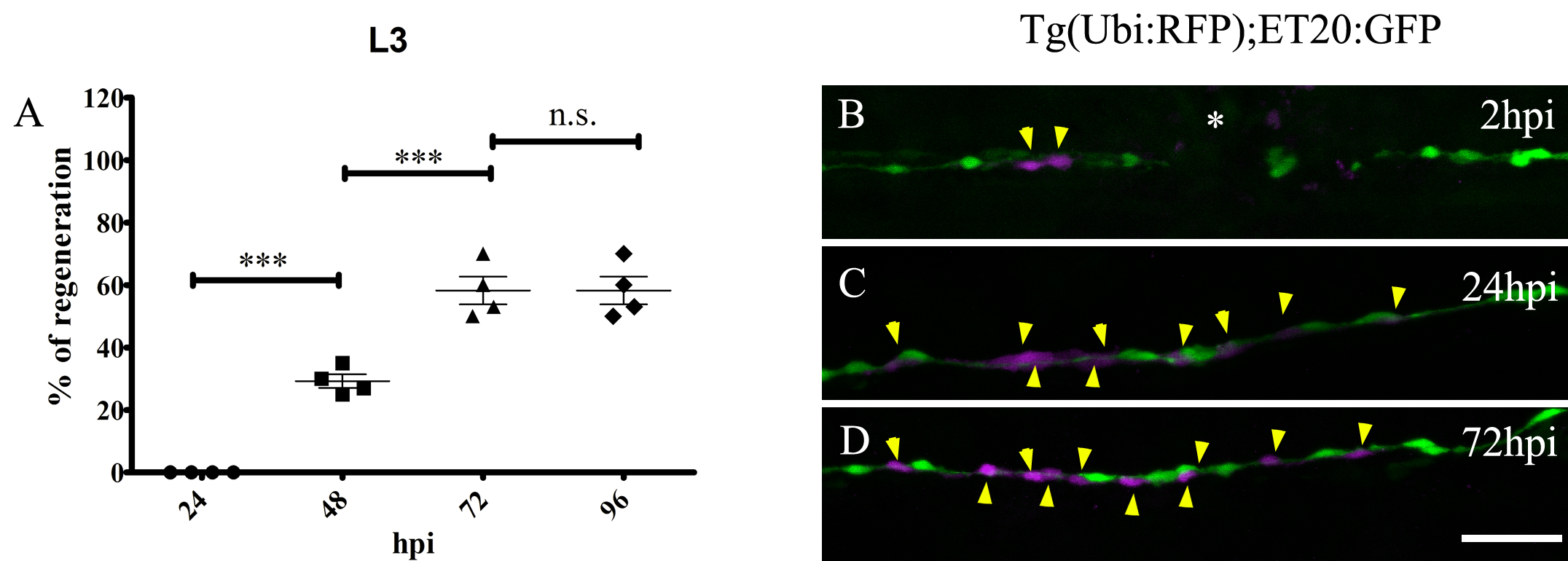

Supplement: Additional file 1: — The non-regenerative outcome of neuromast electroablation results in restoration of a line of INCs at the site of injury. The L3 neuromast of transgenic tg(cxcr4b:mCherry; brn3c:Gap43-GFP) larvae was electroablated and the outcome was scored as regeneration if at least two Bn3c+ cells (hair cells) appeared in the injury zone after the indicated time (n = 100). (A) Quantification of the result; the data are expressed as the percentage of larvae that regenerated the L3 neuromast at different time points after injury. The first regenerated neuromasts can be detected starting from 48 hpi in 29.2 ± 2.2 % of cases. This percentage increases significantly and reaches 58.2 ± 4.4 % after 72 hpi. From 72 to 96 hpi, the percentage of larvae that regenerate neuromasts remains unchanged. (B–D) A 3 dpf tg(et20:GFP) fish was transplanted at the blastula stage with cells from a tg(ubiquitin:RFP) donor fish (transplanted cells pseudocolored in magenta) and its L3 neuromast was electroablated (asterisk in B). In 40 % of cases, the INCs migrate to the injury site, proliferate, but fail to accumulate and organize into a mature neuromast. Yellow arrowheads show the location of the different transplanted cells through time. Scale bar B–D: 50 μm. Further details on replicates are provided in “Quantifications and statistical analysis.” (TIF 4880 kb) [file 12915_2016_249_MOESM1_ESM.tif]

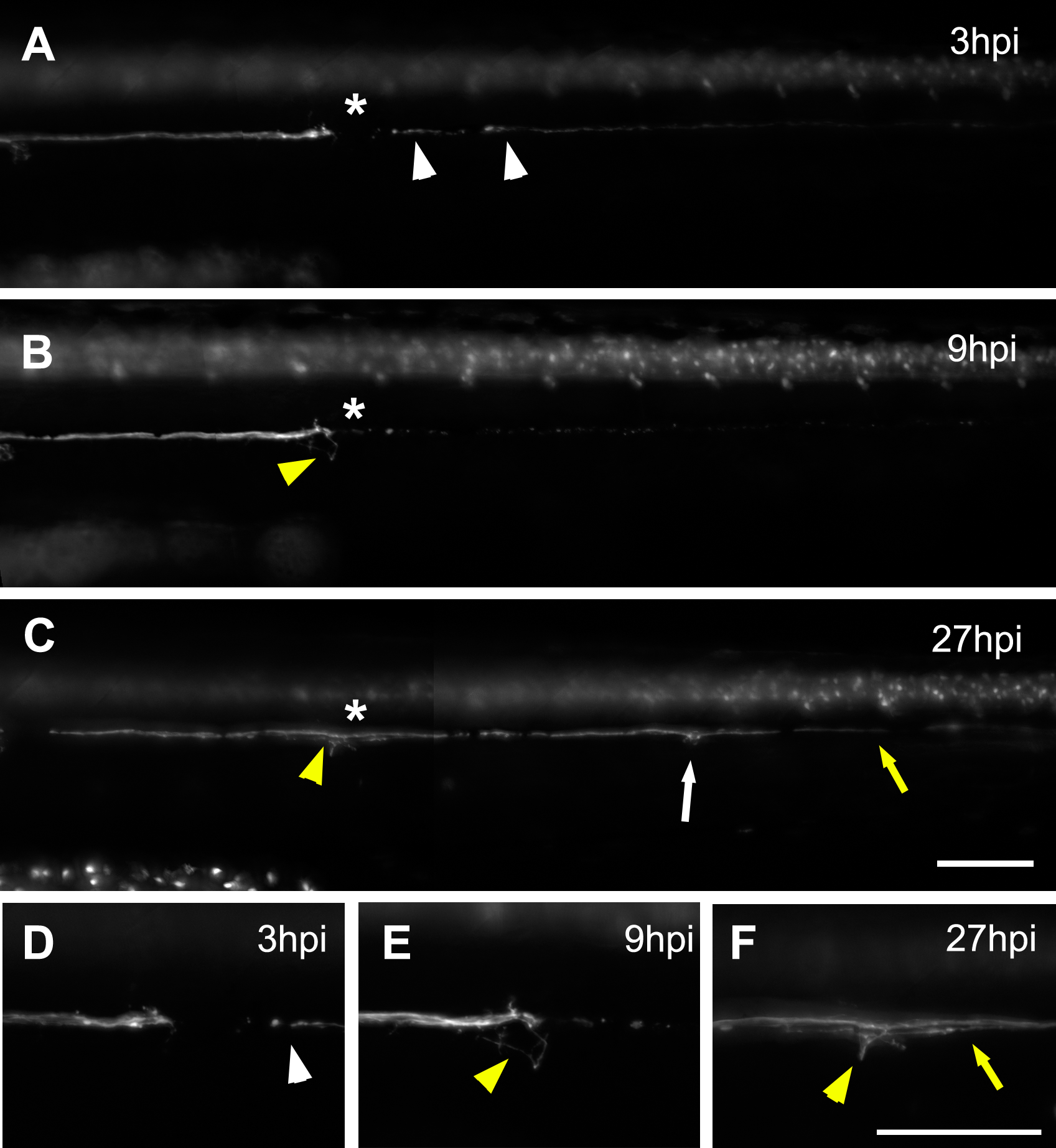

Supplement: Additional file 2: — Behavior of the lateral line nerve after neuromast electroablation. g(neurod:GFP; cxcr4b:mCherry) larvae that express mCherry in all cells types of the lateral line and GFP in the lateral line nerve were electroablated at 3 dpf in the L3 neuromast (asterisk); images show only the GFP channel. (A) At 3 hpi, degenerating fragments of denervated axons are still present at the myoseptum (white arrowheads). (B) At 9 hpi, nerve fragments have been cleared and axons sprouting from the nerve stump begin to extend and explore the injured region (yellow arrowhead). (C) From 9 to 27 hpi, the nerve regrows caudally (yellow arrow). The asterisks indicates the injury site and the yellow arrowhead shows axons innervating this region. Also, the regrowing axons innervate more caudal neuromasts (white arrow). (D–F) Higher magnification of the injury zone at 3 hpi (D), 9 hpi (E), and 27 hpi (F). Scale bar: 100 μm. (TIF 9198 kb) [file 12915_2016_249_MOESM2_ESM.tif]

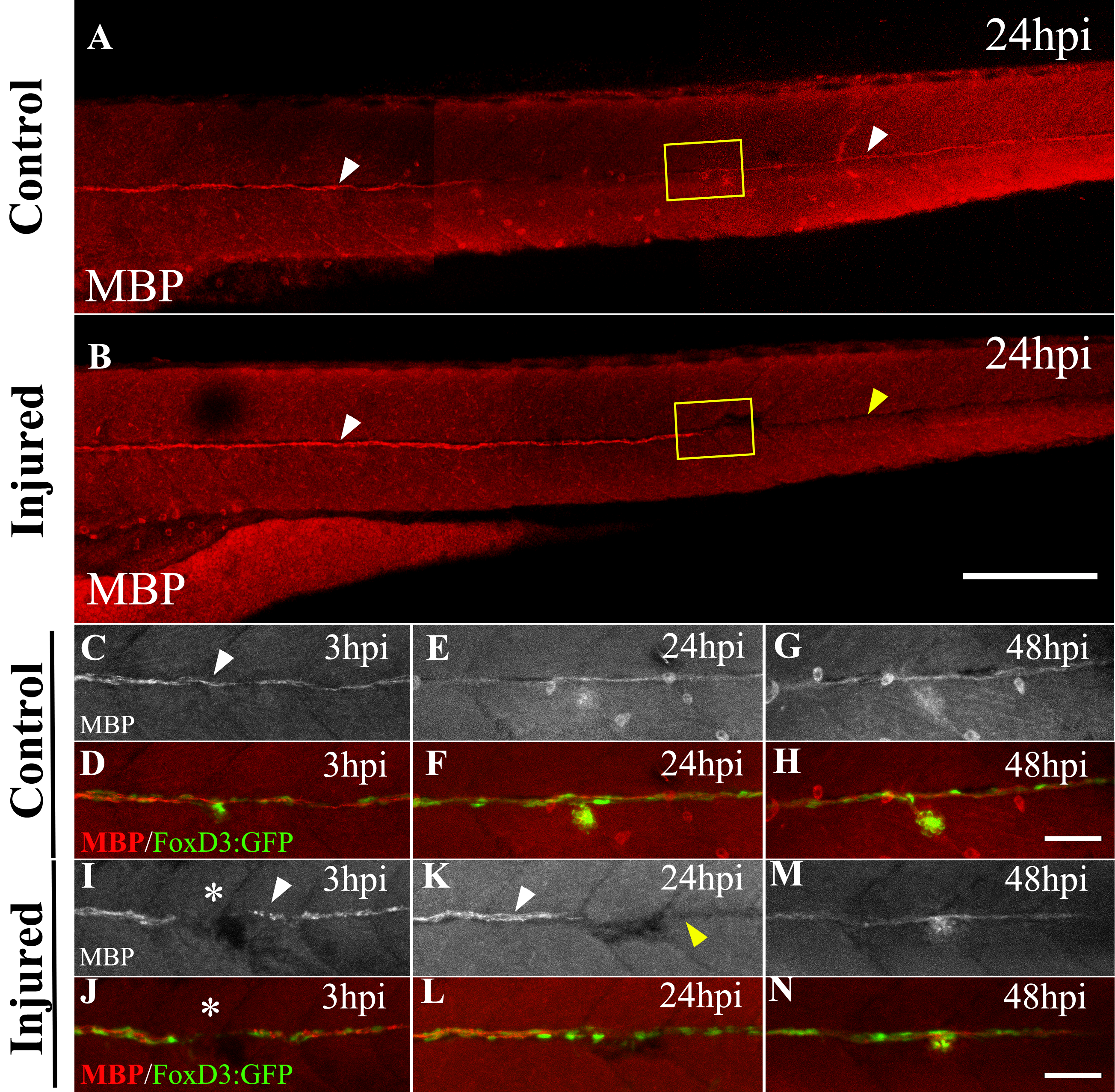

Supplement: Additional file 3: — Temporary loss of MBP expression in caudally located SCs after L3 electroablation. tg(foxd3:GFP; brn3c:GFP) larvae 3 dpf were injured at the L3 neuromast and fixed at different time points. Expression of Brn3c:GFP was used as an indicator to locate the L3 neuromast. Fixed control and injured larvae were processed by immunohistochemistry to analyze MBP and GFP expression. (A, B) White arrowheads show MBP signal in 4 dpf control larvae and 24 hpi in injured larvae (only rostral to L3). The yellow arrowhead in B shows the loss of MBP expression in the caudal zone with respect to the injury site. (C–N) Double transgenic larvae tg(foxd3:GFP; brn3c:GFP) were processed in order to describe, by immunodetection, the time course of MBP expression in the vicinity of L3 in control larvae (C–H) vs injured larvae (I–N). MBP expression by itself is shown in monocolor (C, E, G, I, K, M), while MBP expression (red) merged with the FoxD3 expression (green) is shown in color (D, F, H, J, L, N). In control larvae, there is continuous expression of MBP from 3 to 5 dpf. The arrowhead in C shows the localization of the L3 neuromast. In injured larvae, the expression of MBP is fragmented after 3 hpi (white arrowhead in I), disappears after 24 hpi caudal to L3 (yellow arrowhead in K), and reappears after 48 hpi (M). Note that, despite the transient loss of the differentiation marker, SCs revealed by the presence of GFP are present throughout the regeneration process (J, L, and N). The asterisk in I and J indicates the site where injury was made. Scale bar A, B: 200 μm, C–N: 50 μm. (TIF 6575 kb) [file 12915_2016_249_MOESM3_ESM.tif]

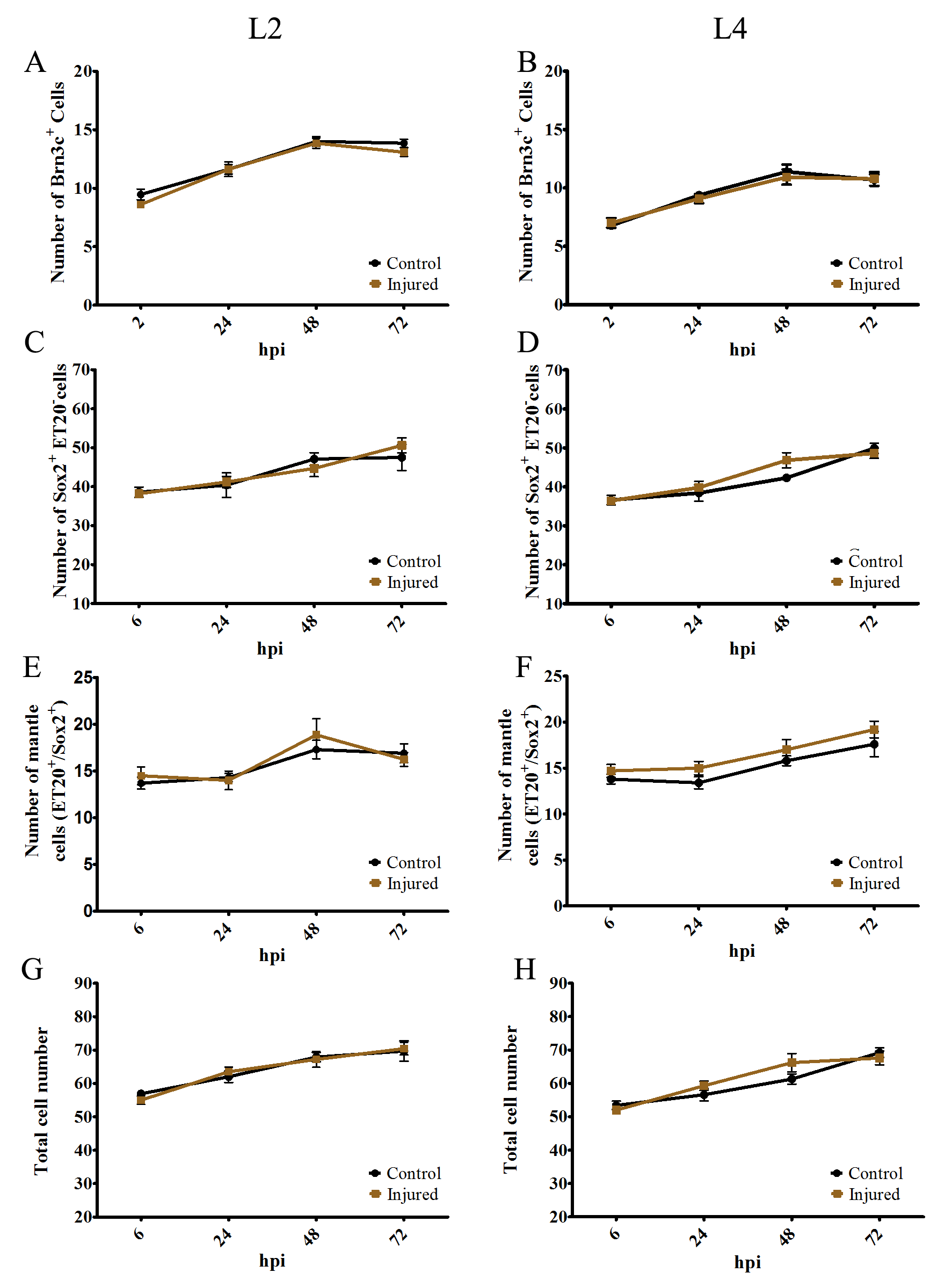

Supplement: Additional file 4: — Electroablation of a neuromast does not affect the cellular composition of neighboring neuromasts. Triple transgenic tg(cxcr4b:mCherry; brn3c:Gap43-GFP; et20:GFP) larvae were electroablated 3 dpf in order to injure the L3 neuromast. Control (black lines) and injured larvae (brown lines) were then processed to determine the cellular composition of neighboring neuromasts located rostral and caudal to the injury zone (L2 and L4, respectively). (A, B) In vivo quantification of Brn3c+ hair cells in the L2 (A) and L4 (B) neuromasts (n = 13) at 2, 24, 48, and 72 hpi in control (black line) and injured larvae (brown line). In all subsequent experiments, quantification times are 6, 24, 48 and 72 hpi. (C, D) Quantification of progenitor cells (Sox2+ET20–) in the L2 and L4 neuromasts (n = 12). (E, F) Double Sox2+ and ET20+ cells were detected by double immunostaining (n = 12). (G, H) The total cell number in neuromasts was quantified by TO-PRO-3 staining. Only cells located within the typical ring structure of the neuromast were counted (n = 12). There are no changes in L2 or L4 neuromast composition after L3 damage. Further details on replicates are provided in “Quantifications and statistical analysis.” (TIF 159 kb) [file 12915_2016_249_MOESM4_ESM.tif]

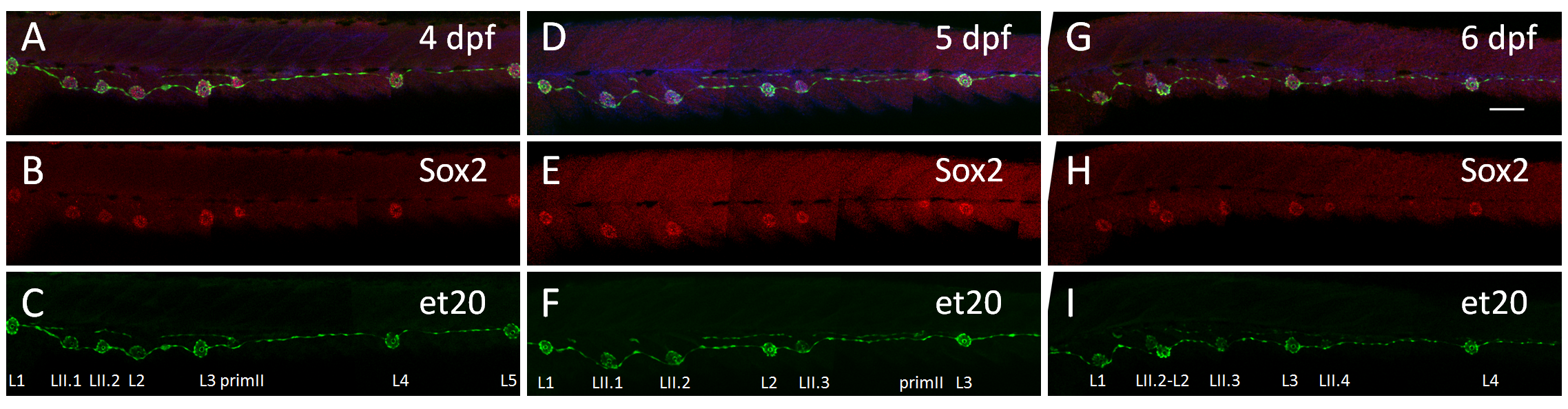

Supplement: Additional file 6: — INCs do not express Sox2 protein. tg(et20:GFP) transgenic larvae at 4, 5, and 6 dpf were fixed and immunolabeled with the anti-Sox2 antibody, revealed with red fluorescence. The top row (A, D, G) shows both GFP signal and Sox2 immunostain; middle row (B, E, H), Sox 2 expression; and bottom row (C, F, I), GFP label. Transgene-driven GFP expression is seen in INCs and in mantle cells of neuromasts. Sox2 expression is only detected in neuromasts, in progenitor cells that underlie hair cells, and in mantle cells (see [33]). At these stages, both primary (L1, L2, etc.) and secondary (LII.1, LII.2, etc.) neuromasts are present and the advancing PrimII (also expressing Sox2) can be seen. (TIF 5802 kb) [file 12915_2016_249_MOESM6_ESM.tif]

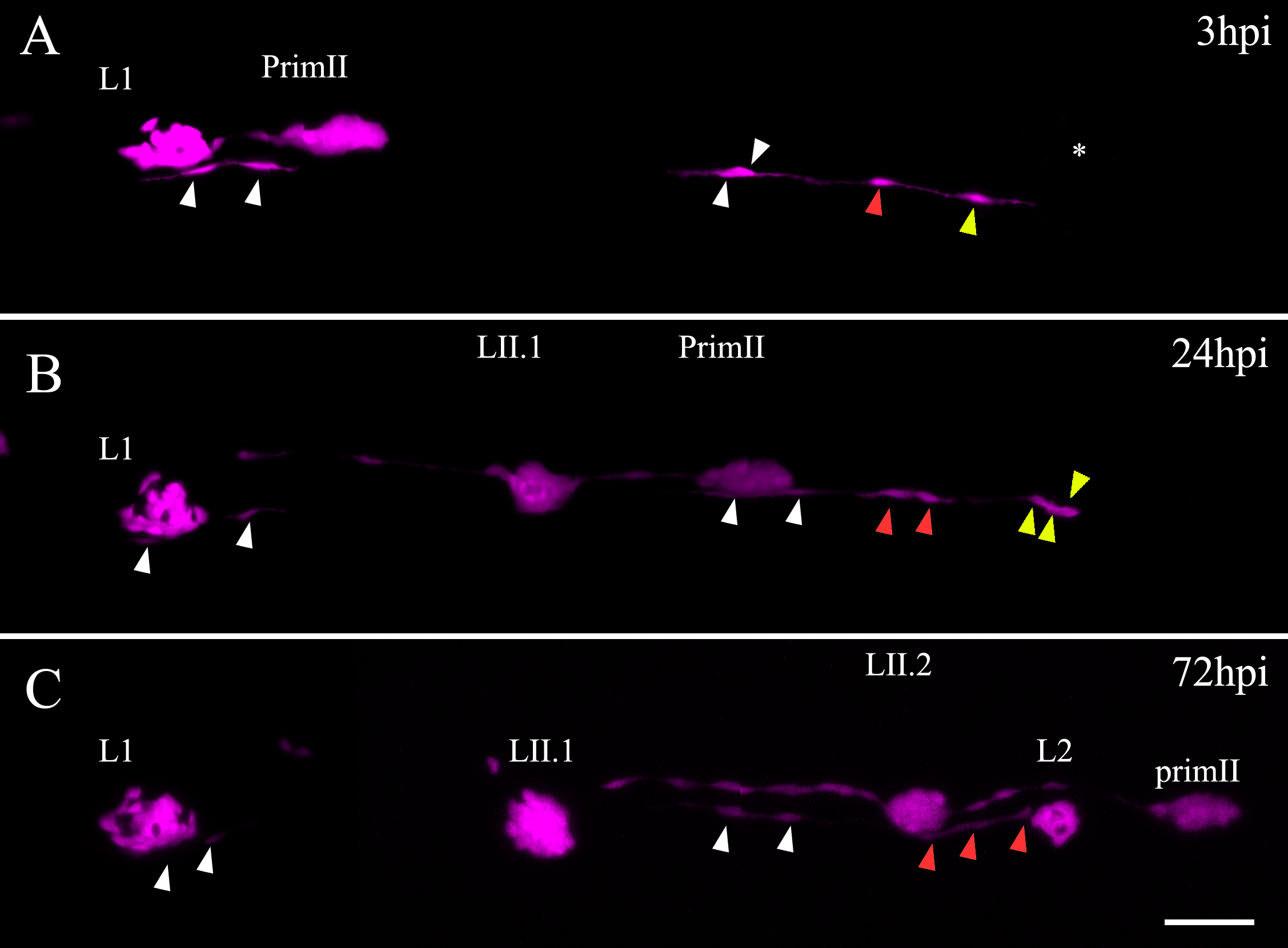

Supplement: Additional file 8: — INCs respond to damage and behave like multipotent progenitor cells. Blastula cells from Tg(ubiquitin:RFP) embryos transplanted into tg(et20:GFP) embryos result in chimeric larvae with a few labeled INCs (magenta, labeled with arrowheads) that can be followed through time in vivo. (A) At 3 dpf, a chimeric larva was electroablated in the L2 neuromast; the asterisk shows the injury site. A proximal (yellow arrowhead) and more distal (red and white arrowheads) labeled INCs can be observed. (B) After 24 hpi, the INC proximal to the injury zone has proliferated and daughter cells begin to accumulate (yellow arrowheads) whereas the nearest neighboring INC seen in A divides once but the daughter cells do not move from their position (red arrowheads). The farthest labeled INCs (white arrowheads) do not divide. (C) After 72 hpi, a regenerated L2 neuromast appears in the position where the injury was made. With the exception of two cells, the entire regenerated L2 neuromast is derived from the single labeled INC cell (yellow arrowhead in A). The distal INC has divided once more but does not contribute to the regenerated neuromast (red arrowheads). From 24 hpi to 72 hpi (which corresponds to 4–6 dpf), the PrimII travels through this region and deposits the secondary neuromasts LII.1 and LII.2. Scale bar: 50 μm. (TIF 1069 kb) [file 12915_2016_249_MOESM8_ESM.tif]

**A**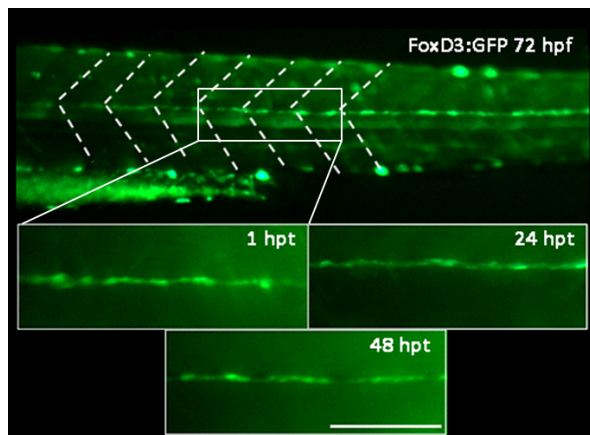**B**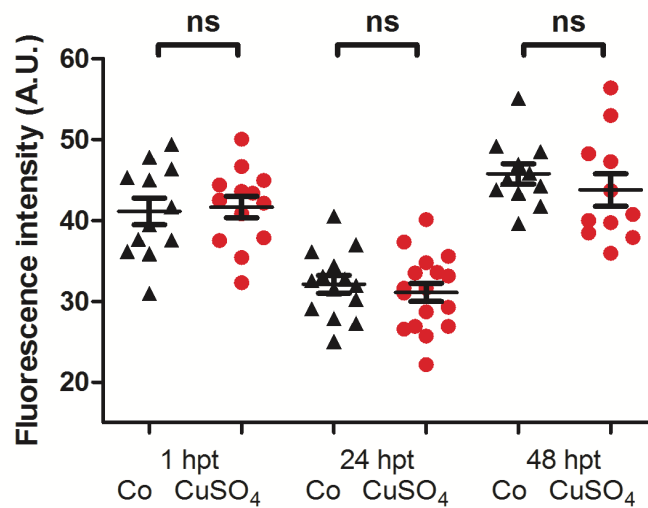**C**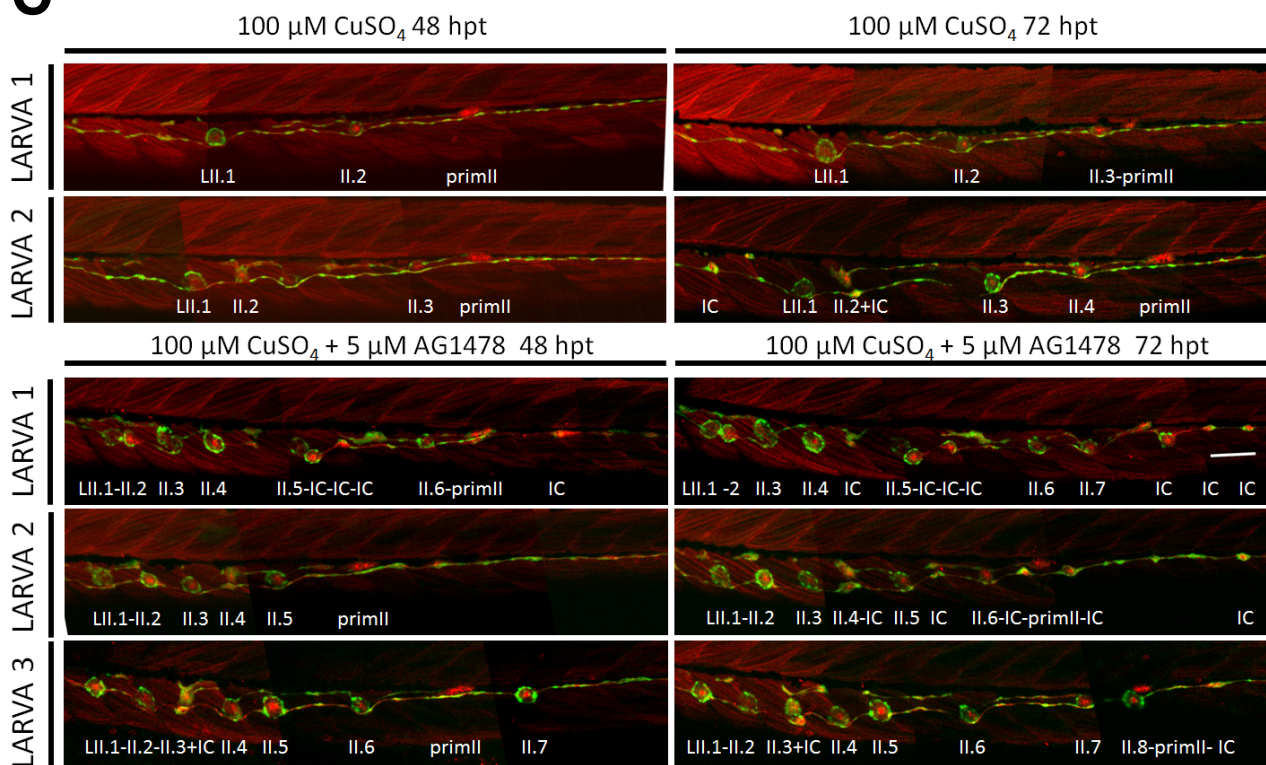

Supplement: Additional file 10: — Copper sulfate treatment does not affect SCs or INCs. (A, B) 72 hpf tg(foxd3:GFP) transgenic larvae (SCs labeled with GFP) were treated for 2 h with 100 μM CuSO4 and imaged 1, 24, and 48 hpt; sibling control fish were left untreated. (A) An arbitrary area of the trunk above the anus was defined (encompassing the width of about three somites) and the “Measure Tool” of ImageJ was used to assign arbitrary units for mean green fluorescence intensity. (B) The graph shows data for control (Co) and copper-treated (CuSO4) fish at the three selected times. While mean values are significantly different between the developmental stages, control fish vs CuSO4-treated fish (control 1, 24, 48 h n =12, 14, 11 respectively and 1, 24, 48 hpt n =13, 17, 11 respectively) at equivalent ages were not significantly different (ns) (P < 0.05). (C) Double transgenic tg(cxcr4:mCherry; et20:GFP) larvae at 3 dpf were treated for 2 h with 100 μM CuSO4 and imaged 48 and 72 hpt. Two larvae are shown at both time points. Note that only secondary lateral line neuromasts (LII) are seen because the copper treatment at day 3 permanently ablates all primary neuromasts. In the bottom three rows, sibling fish were treated in the same way but, after the copper solution was washed out, 5 μM AG1478 was added to the medium and the fish were imaged 48 or 72 hours later; three larvae are shown at both time points. Note that supernumerary neuromasts appear in these animals (compare with two larvae in top half), indicating that INCs are not affected by copper treatment and retain their progenitor potential. (PDF 1994 kb) [file 12915_2016_249_MOESM10_ESM.pdf]
